# ALFQ adjuvanted HIV-1 envelope protein vaccination elicits durable functional antibody and cellular responses in nonhuman primates

**DOI:** 10.1038/s41541-025-01322-7

**Published:** 2025-12-19

**Authors:** Shikha Shrivastava, Joshua M. Carmen, Jiae Kim, Kristina K. Peachman, Shraddha Basu, Ryan Alving, Danielle Nettere, Gautam Kundu, Lauren Yum, Mohammad Arif Rahman, Shalini Jha, Hung V. Trinh, Lorean Rosado, Phuong Nguyen, Elaine Morrison, Isabella Swafford, Kawthar Machmach, Jessica S. Bolton, Adam Yates, Elina Misicka, Zoltan Beck, Simona Mutascio, Ousman Jobe, Michelle Zemil McCrea, Lindsay Wieczorek, Elke S. Bergmann-Leitner, Victoria Polonis, Dominic Paquin-Proulx, Justin Pollara, Gary R. Matyas, Carl R. Alving, Rasmi Thomas, Genoveffa Franchini, Guido Ferrari, Barton F. Haynes, Julie A. Ake, Mangala Rao

**Affiliations:** 1 US Military HIV Research Program, Walter Reed Army Institute of Research, Silver Spring, MD USA; 2 Center for Infectious Disease Research, Walter Reed Army Institute of Research, Silver Spring, MD USA; 3 Henry Jackson Foundation for the Advancement of Military Medicine Inc, Bethesda, MD USA; 4 Oak Ridge Institute of Science and Education, Oak Ridge, TN USA; 5 Duke University Medical Center, Duke Human Vaccine Institute and Department of Surgery, Durham, NC USA; 6 Animal Models and Retroviral Vaccines Section, Basic Research Laboratory, Center for Cancer Research, National Cancer Institute, Bethesda, MD USA; 7 Agile Vaccines and Therapeutics, Navy Medical Research Center, Silver Spring, MD USA; 8 Biologics Research and Development Branch, Walter Reed Army Institute of Research, Silver Spring, MD USA; 9 Duke University School of Medicine, Duke Human Vaccine Institute, Departments of Medicine and Integrative Immunobiology, Durham, NC USA; 10 Present Address: Viral Vaccines Branch, Pfizer; Pearl River, New York, NY USA; 11 Present Address: Technical Integration and Defense Division, Pentagon Force Protection Agency, Pentagon, VA USA; 12 Present Address: Diagnostics and Countermeasures Branch, Walter Reed Army Institute of Research, Silver Spring, MD USA

**Keywords:** Immunology, Diseases

## Abstract

Adjuvants play an important role in modulating antigen-specific immune responses. We conducted a comparative adjuvant immunogenicity study in Rhesus macaques using HIV-1 subtype B gp120 envelope protein, B.63521, formulated with aluminum hydroxide gel (AH), or a family of liposomal adjuvants known as Army Liposome Formulation (ALF). ALF comprises saturated phospholipids, cholesterol, and monophosphoryl lipid A. Inclusion of QS-21 or adsorption of the antigen to AH, followed by the addition of ALF, generates ALFQ and ALFA, while inclusion of both immunostimulants generates ALFQA. Priming with canarypox vector ALVAC, followed by boosting with ALVAC and gp120 formulated with each of the four adjuvants, resulted in ALFQ and ALFQA outperforming AH and ALFA vaccine formulations with a high frequency of antigen-specific plasma cells in the bone marrow, robust antibodies, and Env-specific polyfunctional CD8^+^ T cell responses. Transcriptomic analyses revealed upregulation of antiviral and innate immune pathways, thus highlighting ALFQ as a highly potent adjuvant.

## Introduction

Development of an efficacious vaccine against HIV-1 has been the goal of scientists since the discovery of HIV-1 as the causative agent of acquired immunodeficiency syndrome (AIDS). Despite the progress made in HIV-1 prevention and treatment and several breakthroughs in understanding viral pathogenesis, a vaccine against HIV-1 remains elusive. As of 2024, according to the Global HIV and AIDS statistics fact sheet^1^, 40.8 million people were living worldwide with HIV-1, with 1.3 million new infections, and 630,000 deaths from AIDS-related illnesses in 2024. Numerous studies conducted in nonhuman primates (NHPs) with different vaccine strategies and vector platforms have shown partial protection against SIV or SHIV acquisition^2–5^. The field has oscillated between developing vaccines that target T cell responses and antibodies, resulting in the failure of several clinical trials. The current thinking is that for a vaccine to be maximally efficacious, it must induce potent and broadly neutralizing antibodies. This concept is partially based on the AMP trial^6^, the non-efficacy and discontinuation of the South African HVTN 702 trial, and the failed 705 and 706 trials^7,8^. Thus far, RV144^9^ is the only clinical trial out of a total of nine phase 3 clinical trials conducted^9,10^, that demonstrated a staggering 60% efficacy at 6 months that declined to a still significant 31.2% efficacy against viral acquisition at 36 months, the former a post hoc analysis^9,11^. The RV144 vaccine regimen consisted of ALVAC prime followed by boosting with ALVAC and bivalent HIV-1 Env gp120 proteins formulated in aluminum hydroxide. V1V2-specific IgG binding antibodies were identified as the primary correlate of protection from HIV-1 acquisition. Antibody-dependent cellular cytotoxicity (ADCC), antibody-dependent cellular phagocytosis (ADCP), IgG antibody avidity, polyfunctional envelope-specific CD4^+^ T cells, and cytotoxic CD8^+^ T cells were identified as secondary correlates of reduced risk of HIV-1 acquisition^12–14^. Notably, neutralizing antibodies were not associated with protection and had the highest odds ratio for viral acquisition. As a follow up, several novel platforms including mRNA-LNPs^15,16^, germline targeting strategies^17^, improved gp140 Env trimers with repair and stabilization^18^, and use of novel adjuvants have shown promise in animal studies and are being transitioned to phase 1 clinical trials^19–21^.

We have developed a family of liposomes, which are phospholipid vesicles, known as the Army Liposome Formulation (ALF)^22^. In addition to the presence of synthetic monophosphoryl lipid A, 3D-PHAD™ in ALF, a protein can first be adsorbed to an immunostimulant, such as an aluminum salt and then added to ALF to generate ALFA. Addition of another immunostimulant, the saponin QS-21, either to ALF or to ALFA, generates ALFQ and ALFQA, respectively. Thus, the ALF formulations can contain single or multiple immunostimulants and serve as robust adjuvants for vaccines. Several licensed vaccines, Shingrix®, Arexvy, and Mosquirix that contain QS-21 (AS01B) or Novavax’s COVID vaccine that contains a mixture of QS saponins (Matrix-M^™^) as adjuvants have proven to be highly efficacious^23–26^. ALFQ, like AS01, is a liposome formulation and contains two immunostimulants, MPLA and QS-21. However, ALFQ differs from AS01B in that it contains 55%, rather than 33% cholesterol; the phospholipid fatty acyl chains of ALFQ are saturated rather than unsaturated; ALFQ contains four times the amount of MPLA vs native lipid A and twice the amount of QS-21 than AS01B in 1 mL of the vaccine; and ALFQ comprises heterogeneous particles, with diameters ranging from 50 nm to ≥30 μm, compared to ~100 nm nanoparticles in AS01B^27^.

Members of the ALF family of adjuvants have been successfully tested in multiple vaccine clinical trials against various infectious diseases and cancer^28^, and ALFQ has been used in numerous small animals and NHP immunogenicity studies^16,29–31^ and in several phase 1 vaccine clinical trials^32,33^. Additional phase 1 vaccine clinical trials that utilize ALFQ are currently being evaluated for safety and immunogenicity, including an ongoing HIV-1 vaccine trial containing subtypes B (B.63521) and CRF01_AE (A244) proteins formulated with three different doses of ALFQ (Clinical trial number #NCT05423418).

In the present study, we evaluated the ALF family of adjuvants, ALFA, ALFQ, and ALFQA and the standard and widely used aluminum salt adjuvant, aluminum hydroxide (Alhydrogel, AH), in a comparative vaccine-adjuvant immunogenicity study with HIV-1 subtype B gp120 envelope protein in NHPs. The RV144 regimen, originally designed to protect against HIV-1 subtypes B/E strains, was adapted to incorporate subtype C antigens in ALVAC and subtype B gp120 protein adjuvanted with the various adjuvant formulations mentioned above. We assessed the immunogenicity of the four vaccine-adjuvant formulations to determine if inclusion of more than one immunostimulant improved the cellular, humoral, and functional immune responses, and induced specific gene signatures. The QS-21-containing vaccine-adjuvant formulations (ALFQ and ALFQA) elicited strong and durable binding antibody responses along with robust neutralizing and Fc-mediated functional humoral immune responses. ALFQ and ALFQA also induced higher percentages of Env-specific polyfunctional CD4^+^ and CD8^+^ T cell responses, including IL-21-secreting T follicular helper cell (Tfh) responses and long-lived antigen-specific plasma cells (LLPCs) in the bone marrow. Gene set enrichment analysis (GSEA) identified distinct gene signatures associated with innate immune and antiviral pathways that were specific to QS-21-containing vaccine formulations. A monocyte signature associated positively with the frequency of Env-specific CD8^+^ T cells expressing IFN-γ in the QS-21 saponin adjuvant groups (ALFQ + ALFQA). The NHP data further bolster the potency of ALFQ and ALFQA as vaccine adjuvants.

## Results

### Immunization scheme, safety, and reactogenicity in Rhesus macaques

**Fig. 1:**
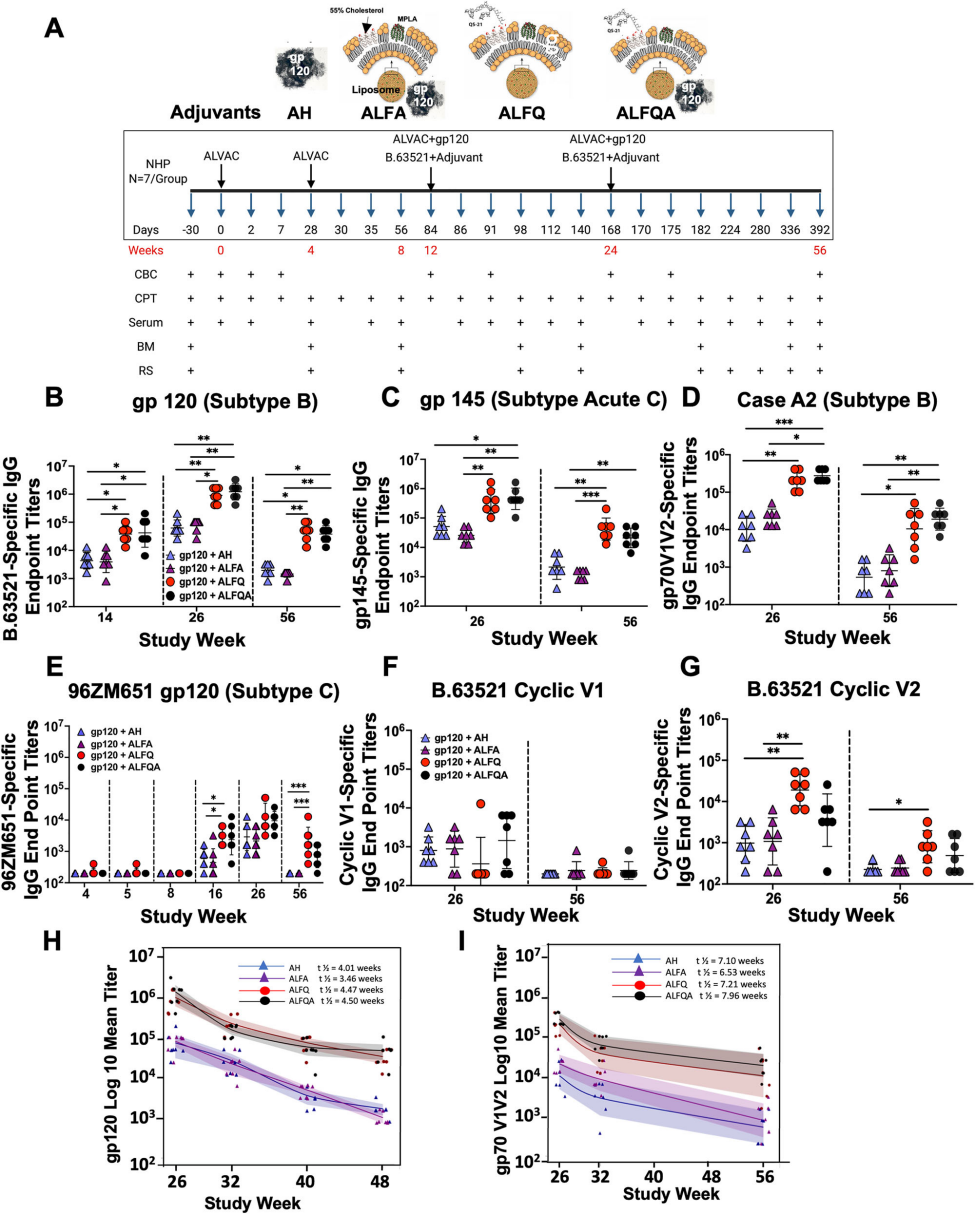
Binding antibody titers in Rhesus macaques. **A** Rhesus macaques (*N* = 7/group) were primed at weeks 0 and 4 with canarypox vector ALVAC vCP2438 (1.14 × 10^7^ pfu) and then boosted at weeks 12 and 24 with ALVAC vCP2438 and HIV-1 Env gp120 protein, B.63521 adjuvanted with Alhydrogel®, ALFA, ALFQ, or ALFQA. Specimens were collected at various timepoints as shown. **B**–**G** Binding antibody titers for each of the four groups AH: blue triangles; ALFA: purple triangles, ALFQ: red circles, ALFQA: black circles were quantified as ELISA endpoint titers against **B** B.63521 gp120, **C** gp145 acute C, **D** gp70V1V2 Case A2, **E** 96ZM651 gp120 (subtype C) proteins, **F** B.63521 cyclic V1 peptide, and **G** B.63521 cyclic V2 peptide. **H**, **I** Antibody geometric mean decay and half-life following vaccination. The log10-transformed geometric mean titer decay, 95% upper and lower confidence bounds for the mean, and per-NHP distribution within each timepoint across the study weeks and the titers for the four groups are depicted for **H** gp120 and **I** gp70V1V2. Data points for each graph indicate endpoint titers observed for each NHP at a given timepoint; line and bands indicate each titer’s geometric mean value and upper and lower confidence limits around the mean. Each dot in all the panels represents the average for triplicate measurements from each animal at the timepoints indicated. Statistical comparisons between different adjuvant groups were performed using the Kruskal–Wallis test with Dunn’s multiple comparison test. *p < 0.04, **p < 0.009, and ***p < 0.0007.

Four groups of Rhesus macaques (*N* = 7/group) received two priming vaccinations with canarypox vector ALVAC vCP2438 followed by two boosting vaccinations comprising ALVAC vCP2438 (1.14 × 10^7^ pfu) and HIV-1 Env gp120 subtype B protein, B.63521 (100 µg) formulated with AH (300 µg Al^3+^), or liposomal formulations: ALFA (100 µg MPLA and 300 µg Al^3+^), ALFQ (100 µg MPLA and 50 µg QS-21), or ALFQA (100 µg MPLA, 50 µg QS-21, and 300 µg Al^3+^) in a total volume of 500 µL. Blood, bone marrow, and rectal secretions were collected at the timepoints shown in Fig. 1A. The vaccinated animals were closely monitored for adverse events during the study. There were no local reactions at the vaccination site. No major vaccine-related differences in body weight, temperature, respiration, or pulse were observed among the four groups (Fig. S1A–D).

The plots of group averages for blood chemistry for the 4 groups are shown in Fig. S2. There were no significant elevations in biomarkers for kidney (creatinine and urea) or liver (Bilirubin, AST, ALT, ALKP, and Albumin) function following vaccination. The blood cell and hematological cell counts after each of the two protein vaccinations and at the end of the study week, as well as the reference range for each parameter are shown in Tables S1– S4 and were within the range for all the groups throughout the study except for an elevated platelet count in one out of seven NHPs in the ALFQ group, 7 days (study day 91) after the first protein vaccination. Overall, these data demonstrated that there were no major fluctuations in the blood chemistry or blood counts and that all four vaccine-adjuvant formulations were found to be safe and tolerable, and none of them induced any adverse systemic events in any of the animals.

### ALFQ and ALFQA induce robust HIV-1 Env-specific IgG binding antibody responses

The antibody responses two weeks after the first and second protein boosts are shown in Fig. 1B. B.63521 gp120 formulated with ALFQ and ALFQA with the same saponin dose induced significantly higher antigen-specific IgG titers, 2 weeks post-first protein vaccination (week 14), compared to AH (*p* = 0.014) and ALFA (*p* = 0.013) groups containing the same dose of Al^3+^. The responses were further increased 2 weeks after the second protein vaccination at week 26 (Fig. 1B) and were significantly higher compared to AH (*p* = 0.007; *p* = 0.002) and ALFA (*p* = 0.03; *p* = 0.008) groups, respectively. The kinetics of the antibody responses are shown in Fig. S3. Longitudinal analysis of the serum samples demonstrated the peak antibody response to the proteins and the cyclic peptides at week 26 (Figs. S3A–D) for all vaccine groups. The B.63521 gp120-specific antibody responses were 10–15-fold higher at week 26 with ALFQ and ALFQA-vaccines compared to AH and ALFA-vaccines (ALFQ vs AH: *p* = 0.007; ALFQ vs ALFA: *p* = 0.03; ALFQA vs AH: *p* = 0.002; and ALFQA vs ALFA: *p* < 0.008). The antibody responses were durable and maintained at week 56 (8 months after the second protein vaccination) in the ALFQ and ALFQA groups (ALFQ vs AH: *p* = 0.011; ALFQA vs AH; *p* = 0.03; ALFQ vs ALFA: *p* = 0.001; and ALFQA vs ALFA: *p* = 0.004). In contrast, there was a greater than 2-log decrease in the antibody titers in the groups vaccinated with AH and ALFA vaccines (Fig. 1B and Fig. S3A). The endpoint titers for ALFQ and ALFQA at weeks 26 (904,470 and 1.2 × 10^6^) and 56 (46,373 and 38,041), respectively, were higher compared to AH and ALFA at similar timepoints (62, 413, and 76,083 vs. 1950 and 1449). A similar trend was observed with subtype C gp145 (Week 26: ALFA vs ALFQA: *p* = 0.001; ALFA vs ALFQA: *p* = 0.004; and ALFQA vs AH: *p* = 0.033) (Week 56: ALFQ vs AH: *p* = 0.009; ALFQ vs ALFA: *p* = 0.0005; and ALFQA vs ALFA: *p* = 0.007) and subtype B gp70V1V2 Case A2-specific IgG antibodies at weeks 26 (ALFQ vs AH: *p* = 0.004; ALFA vs ALFQA: *p* = 0.013; and ALFQA vs AH: *p* = 0.0006) and 56 (ALFQ vs AH: *p* = 0.014; ALFA vs ALFQA: *p* = 0.009; and ALFQA vs AH: *p* = 0.002), respectively (Fig. 1C, D and Fig. S3B). The CHO-produced HIV Env gp145 C.6980 protein was down-selected from four East African HIV-1 subtype C strains derived from an individual in the acute phase of HIV-1 infection. The recombinant gp70V1V2 fusion protein consists of a C-terminal His-tagged gp70 from murine leukemia virus and V1V2loops from HIV-1 Clade B/Case A2.

The antibody responses against 96ZM651 gp120, the protein encoded by ALVAC vCP2438 was also examined. No antibody responses were induced 4 weeks after the first ALVAC vaccination and 1 week (week 5) after the second ALVAC vaccination (Fig. 1E). 96ZM651 gp120-specific IgG antibody responses were observed in all four groups following administration of the adjuvant. The endpoint titers for the ALFQ group were significantly higher compared to AH and ALFA groups at week 16 (ALFQ vs AH: *p* = 0.05; ALFQ vs ALFA: *p* = 0.014) and week 56 (ALFQ vs AH: *p* = 0.0007; ALFQ vs ALFA: *p* = 0.007), respectively (Fig. 1E). Furthermore, the antibody responses were maintained at week 56 in the ALFQ and ALFQA groups, whereas they were back to baseline in the AH and ALFA groups. The endpoint titers against 96ZM651 gp120 were much lower compared to the endpoint titers against B.63521 gp120 at the timepoints tested, despite 80% identity in the sequences between subtype C 96ZM651 and subtype B B.65321 proteins.

No significant differences (*p* > 0.05) in the IgG antibody responses were observed with the cognate cyclic V1 peptide (Fig. 1F and Fig. S3C). However, the ALFQ-vaccine induced significantly higher antibody responses to cognate V2 peptide at weeks 26 (ALFQ vs AH: *p* = 0.002; and ALFQ vs ALFA: *p* = 0.005) and 56 (ALFQ vs AH: *p* = 0.04) compared to the other groups (Fig. 1G and Fig. S3D).

To determine if the decrease in antibody titers in the AH-containing groups merely reflected a decrease in magnitude, the decay rate was examined for B.63521 gp120 and gp70V1V2 proteins. The log10-transformed geometric mean titer decay, 95% upper and lower confidence bound for the mean, and distribution per-NHP within each timepoint across the four study timepoints and the endpoint titers for gp120 and gp70V1V2 are depicted in Fig. 1H, I, respectively. The half-life for the (non-transformed) titer decay was calculated as a function of change in titer value between weeks 26 and 48 or between weeks 26 and 56 using the exponential decay method. While the decay rates for AH compared to the other groups were not significant, the decay rates for both ALFQ (*t*½ = 4.47 weeks) and ALFQA (*t*½ = 4.50 weeks) were significantly different from ALFA (*t*½ = 3.46 weeks) for the B.63521 gp120 protein (*p* < 0.05 in each case; Fig. 1H and Table S5). In contrast, there were no significant differences in the pairwise decay rates for the gp70V1V2 protein (Fig. 1I and Table S5).

IgG1 and IgG2 antibodies specific to B.63521 gp120 protein were also significantly higher at week 26 with ALFQ and ALFQA compared to AH or ALFA (IgG1: ALFQ vs AH: *p* = 0.04; ALFQ vs ALFA *p* = 0.002; ALFQA vs ALFA *p* = 0.003; IgG2: ALFQ vs AH: *p* = 0.02; ALFQA vs AH: *p* = 0.01; ALFQ vs ALFA: *p* = 0.005; and ALFQA vs ALFA: *p* = 0.003) (Fig. S3E, F). No significant differences (*p* > 0.05) were observed in the ratio of IgG1 to IgG2 (Fig. S3G). Furthermore, although ALFQ showed a trend towards higher antigen-specific IgG antibody responses at weeks 26 and 56 for rectal secretions, these responses were not statistically different between the various adjuvant formulations (Fig. S3H).

### ALFQ and ALFQA induce higher avidity binding antibodies and neutralizing antibodies

**Fig. 2:**
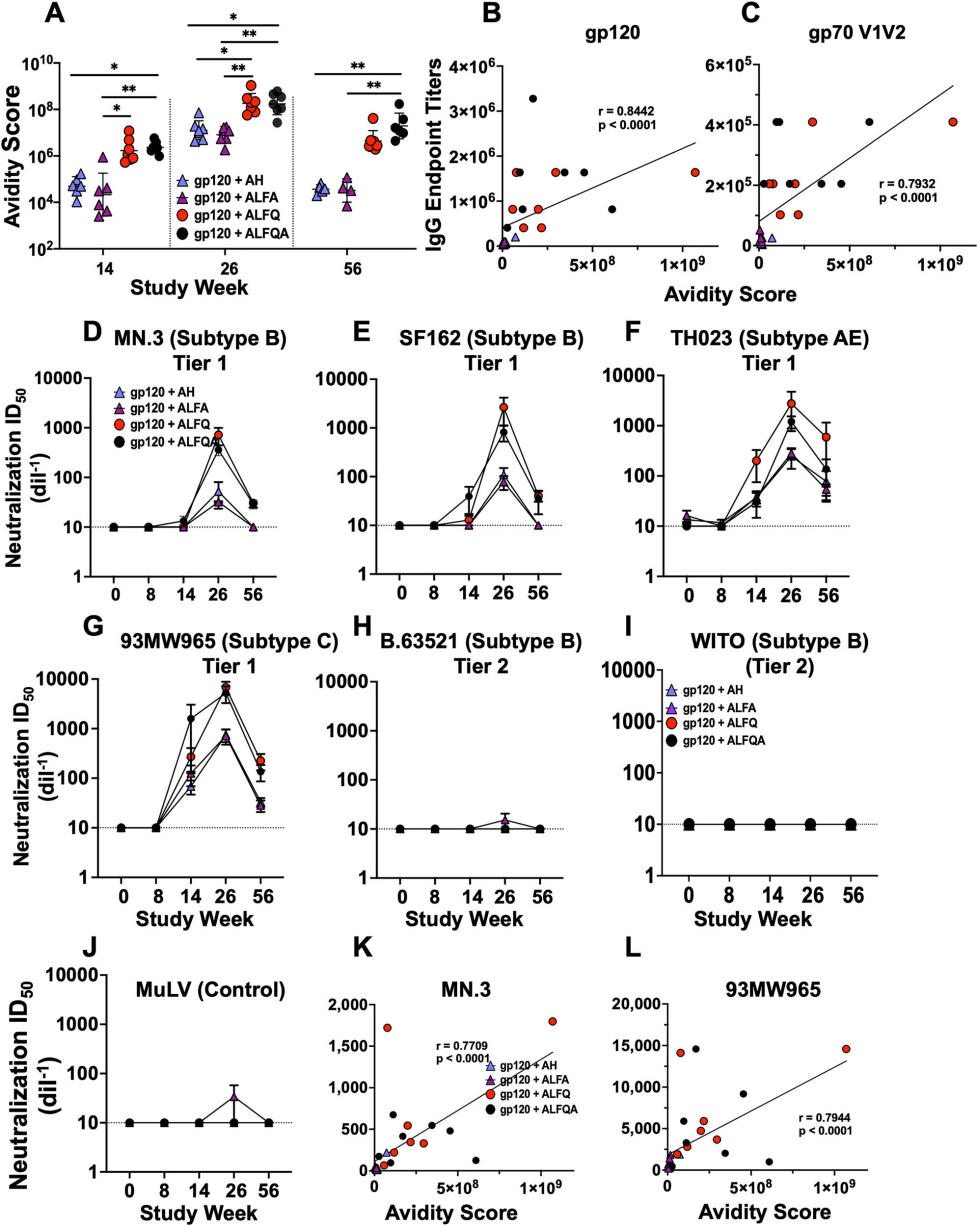
Avidity score correlations and neutralizing antibody titers. **A** Surface plasmon resonance data showing B.63521 gp120 protein-specific antibody avidity score (response unit/*K*_d_ off-rate) at weeks 14, 26, and 56. The data were represented as a geometric mean avidity score with geometric SD. Statistical comparisons between different adjuvant groups were performed using the Kruskal–Wallis test with Dunn’s multiple comparison test. *p < 0.02, **p < 0.008. **B**, **C** Spearman correlation between **B** B.63521 gp120- and **C** gp70V1V2 Case A2-specific IgG endpoint titers and avidity score. Pseudovirus neutralization titers (ID_50_) were measured against **D** MN.3 (tier 1), **E** SF162 (tier 1), **F** TH023 (tier 1), **G** 93MW965 (tier 1), **H** B.63521 (tier 2), **I** WITO (tier 2), and **J** MuLV (negative control). The horizontal black dotted line represents the lower limit of detection. **K**, **L** Spearman correlation between **K** MN.3 and **L** 93MW965 neutralization ID_50_ at week 26 and avidity score are shown.

Individual serum samples were analyzed for B.63521 gp120-specific antibody avidity by surface plasmon resonance-based binding and kinetic analysis. Avidity maturation as depicted by the avidity score (response units/*K*_d_ off-rate) was observed in both ALFQ and ALFQA groups (Fig. 2A) starting at week 14 and was significantly higher, exhibited a 34-fold higher avidity than AH and ALFA (ALFQ vs ALFA: *p* = 0.02; ALFQA vs AH: *p* = 0.01; ALFQA vs ALFA *p* = 0.002) groups, remained significantly higher at weeks 26 (ALFQ vs AH: *p* = 0.02; ALFQA vs AH: *p* = 0.02; ALFQ vs ALFA: *p* = 0.007; ALFQA vs ALFA: *p* = 0.006), and 56, with the avidity score 120-fold higher with ALFQ compared to AH and ALFA vaccines. In contrast, in the AH and ALFA groups, the avidity scores at week 56 were significantly decreased (ALFQA vs AH: *p* = 0.04; ALFQA vs ALFA *p* = 0.008) with the avidity scores at or slightly below the avidity scores observed at week 14 (Fig. 2A). A strong and significant positive correlation was seen between avidity score and binding antibodies against B.65321 gp120 (*r* = 0.8442; *p* < 0.0001) and gp70V1V2 Case A2 (*r* = 0.7932; *p* < 0.0001) proteins (Fig. 2B, C).

Two tier 1 (MN.3, SF162) and two tier 2 (WITO, and B.65321) subtype B, and two tier 1 subtypes CRF01_AE (TH023), and C (93MW965) pseudoviruses, were utilized to evaluate the presence of neutralizing antibodies in the plasma of vaccinated NHPs using the TZM-bl assay (Fig. 2D–I). Murine leukemia virus (MuLV) was used as a nonspecific control (Fig. 2J). Neutralizing antibody responses were not induced after two priming vaccinations with ALVAC but were induced only after the protein boost, which peaked at week 26 (Fig. 2D–G). The responses were tenfold more potent with ALFQ and ALFQA compared to the AH or ALFA formulations. No neutralizing antibody titers were obtained against the two tier 2 subtype B viruses tested, the homologous pseudovirus, B.63521 (Fig. 2H) and WITO (Fig. 2I). A strong and significant positive correlation was observed between avidity score and neutralization against MN.3 (*r* = 0.7709; *p* < 0.0001) and 93MW965 pseudoviruses (*r* = 0.7944; *p* < 0.0001) (Fig. 2K, L).

### ALFQ and ALFQA induce durable Fc-mediated antibody effector functions

**Fig. 3:**
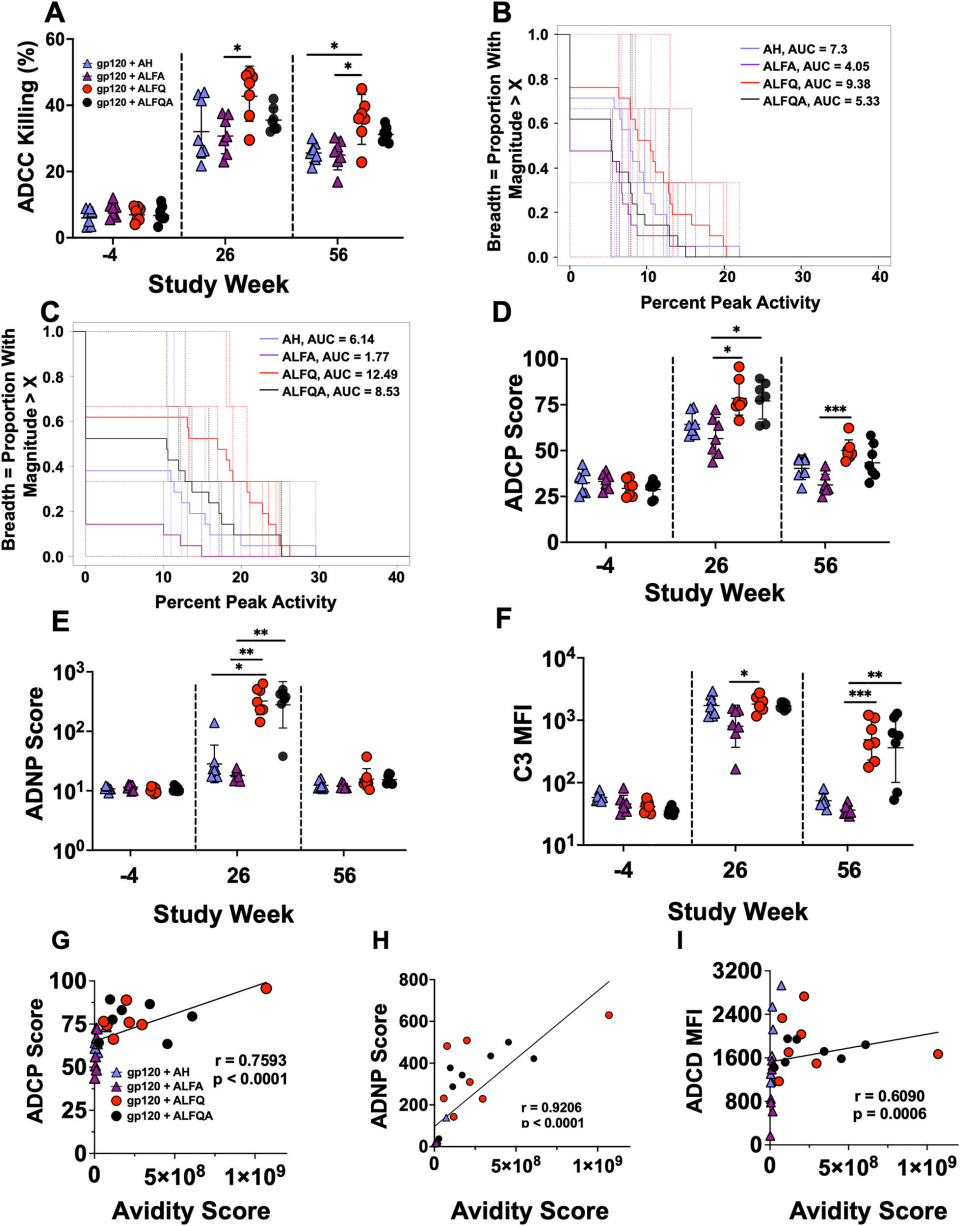
Fc effector functions exhibited by serum antibodies. Plasma samples from individual NHPs for each individual adjuvant formulation was assessed for **A** ADCC with gp120-coated target cells, **B** A magnitude-breadth curve showing the percent peak activity ADCC response and breadth (number of isolates targeted) of an individual sample assayed against three different gp120s for the GTL assay or **C** three different HIV-1 IMCs for the Luc assay using samples from week 26. The x-axis represents the threshold of ADCC responses as peak activity for the three proteins, or three IMCs tested, whereas the y-axis represents the percent of the three targets showing ADCC responses. The AUCs between two groups were compared using Wilcoxon test. In the GTL and LUC assays, the AUC for percent peak activity for ALFQ significantly differed from ALFA group (*p* = 0.0215, p = 0.0061), and from AH group (*p* = 0.0550) in the LUC assay. None of the other groups showed any significance (*p* > 0.4428), **D** ADCP, **E** ADNP, and **F** ADCD. Data were graphed as mean ± SEM. Statistical comparisons between the different adjuvant groups were performed using the Kruskal–Wallis test with Dunn’s multiple comparison test. *p < 0.04, **p < 0.002, and ***p < 0.0008. Spearman correlation between avidity score and **G** ADCP, **H** ADNP, and **I** ADCD for week 26 are shown.

Plasma from weeks -4 (pre-vaccination), 26, and 56 were evaluated for their ability to engage cellular receptors through their Fc region and induce effector functions (Fig. 3). ADCC was assessed using vaccine-matched gp120-coated EGFP-CEM-NKr-CCR5-SNAP target cells that constitutively express GFP (Fig. 3A). There was a significant increase (*p* = 0.0006) in the ADCC activity at week 26 in all groups compared to the responses seen at week -4 (Fig. 3A). Importantly, the ADCC activity was significantly higher with ALFQ compared to ALFA (*p* = 0.04) vaccine at week 26 and at week 56 compared to AH (*p* = 0.01) and ALFA (*p* = 0.02) (Fig. 3A). Inclusion of a third immunostimulant (ALFQA) did not further improve these responses.

The breadth of the ADCC responses at week 26 was determined by utilizing target cells either coated with gp120 or infected with vaccine-matched subtype C 96ZM, heterologous subtypes B WITO, and C TV1 infectious molecular clones (IMC). The results are reported as magnitude-breadth curves used to describe the magnitude of the ADCC responses as peak activity and breadth as the number of isolates targeted by an individual sample using gp120-coated (Fig. 3B) or infected target cells (Fig. 3C). In both assays, the highest ADCC responses were detected only in the ALFQ-vaccine group. Moreover, percent peak ADCC responses were significantly higher in ALFQ compared to ALFA in the gp120-coated GTL assay (*p* = 0.0215) and infected cells LUC assay (*p* = 0.0061), while a trend for significance was noted compared to AH-immunized animals in the LUC assay (*p* = 0.0550). Similar results were observed when the ADCC titers were analyzed. Comparison of area under the curve for the ADCC titers in the gp120-coated GTL and LUC assays showed significantly higher titers in ALFQ compared to ALFA-vaccinated animals (*p* = 0.0152; *p* = 0.0130), respectively (Fig. S4A, B and Tables S6, S7).

Plasma samples from weeks -4, 26, and 56 were evaluated for other Fc effector functions. At week 26, a significant increase in the ADCP activity was observed in ALFQ and ALFQA groups compared to ALFA (*p* = 0.01; Fig. 3D). At week 56, the ADCP responses had declined in all the groups, however the responses were significantly higher in the ALFQ compared to the ALFA group (*p* = 0.0006) (Fig. 3D). Evaluation of the plasma samples for antibody-dependent neutrophil phagocytosis (ADNP) responses showed that ALFQ and ALFQA groups had significantly higher ADNP scores at week 26 compared to AH and ALFA (ALFQ vs AH: *p* = 0.04; ALFQ vs ALFA: *p* = 0.001; ALFQA vs ALFA: *p* = 0.002; Fig. 3E). However, by week 56, the responses were back to baseline in all groups, indicating that these responses were not durable.

At week 26, all groups showed high antibody-dependent complement deposition (C3) ability (ADCD) responses with significantly higher responses with ALFQ compared to ALFA (*p* = 0.04; Fig. 3F). However, by week 56, the responses drastically declined back to pre-immunization levels in the AH and ALFA groups. In contrast, the responses were maintained at high levels in both ALFQ and ALFQA groups and were significantly higher than the ALFA (ALFQ vs ALFA: *p* = 0.0008; ALFQA vs ALFA: *p* = 0.001) group, thus demonstrating that ALFQ and ALFQA vaccine-adjuvant formulations induced durable responses (Fig. 3F). A significant and strong positive correlation between ADCP (*r* = 0.7593; *p* = 0.0001), ADNP (*r* = 0.9206; *p* = 0.000.1) and ADCD (*r* = 0.6090; *p* = 0.0006) responses and avidity score was seen in each case at week 26 (Fig. 3G–I).

### ALFQ and ALFQA induce a higher percentage of gp120-specific long-lived plasma cells (LLPCs) in the bone marrow

**Fig. 4:**
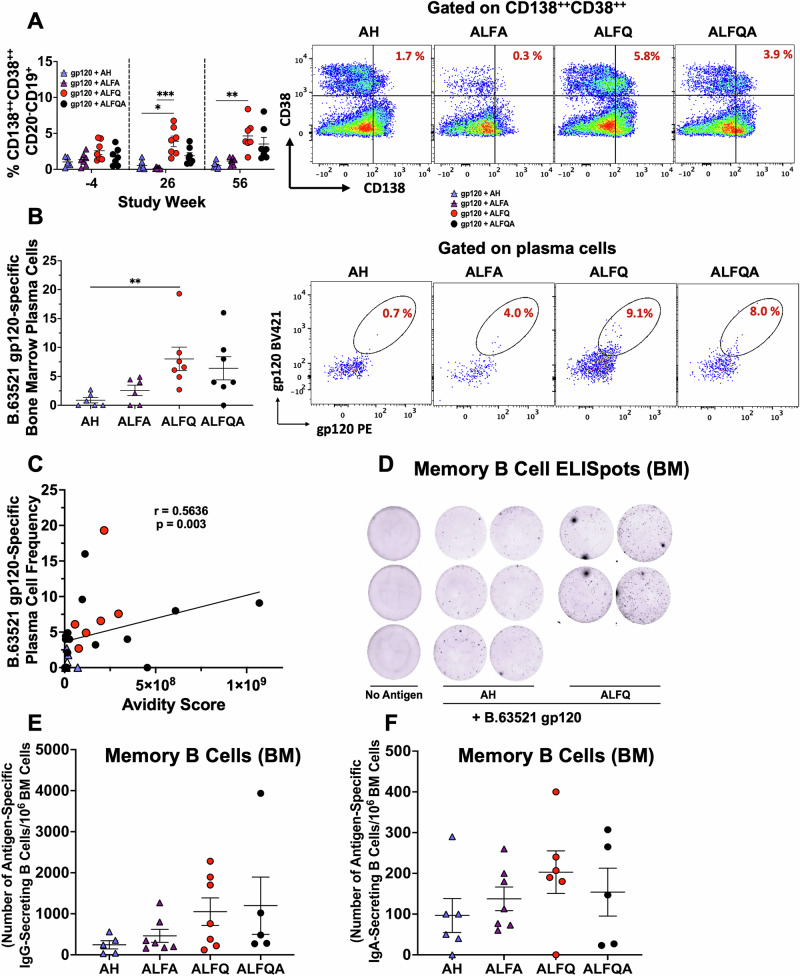
Plasma cells, B.63521 gp120-specific LLPCs, and memory B cells in the bone marrow. **A** The frequency of CD138^++^CD38^++^CD20^-^CD19^+^ plasma cells at weeks -4, 26, and 56 are presented as mean ± SEM. Each individual NHP is represented as a symbol (*N* = 7/group). **B** The percentage of gp120 protein-specific plasma cells in the bone marrows collected at week 56, positive for intracellular dual-fluorophore labeled B.63521 gp120 proteins (gp120-BV421 and gp120-PE). The right panels show the representative flow plots. The flow panel and the gating strategy are shown in Table S8 and Fig. S5A, respectively. **C** Spearman correlation between B.65321 gp120-specific bone marrow plasma cell frequency and antibody avidity score at week 56 is shown. **D** Memory B cell ELISpot images of duplicate or triplicate wells from bone marrow cells from one or two animals in the group, depicted at week 48 following in vitro stimulation of BM cells. The B.63521 gp120 protein-specific IgG memory B cell ELISpot images following stimulation are shown for naïve, AH, and ALFQ groups. **E** The number of memory B cell B.63521 gp120-specific IgG and **F** IgA spot-forming units/10^6^ bone marrow cells are presented as mean ± SEM. Each symbol represents the average of triplicate or duplicate wells for each NHP from each group. The responses were considered positive when the spot count exceeded the mean ± 3 SD of the negative control wells. Statistical comparisons between the different adjuvant groups were performed using the Kruskal–Wallis test with Dunn’s multiple comparison test. *p < 0.01, **p < 0.008, and ***p < 0.0002.

Terminally differentiated LLPCs persist in the bone marrow for long periods of time. Bone marrow cells collected at weeks -4, 26, and 56 were analyzed for the presence of LLPCs (CD138^++^CD38^++^CD20^-^CD19^+^) by flow cytometry^34^. The flow panel and the gating strategy are shown in Table S8 and Fig. S5A, respectively. The right panel shows representative flow plots (Fig. 4A). The frequency of LLPCs were significantly higher in the ALFQ compared to the AH group (ALFQ vs AH: *p* = 0.013) or ALFA (ALFQ vs ALFA: *p* = 0.0002) vaccines at weeks 26 and 56 (ALFQ vs AH: *p* = 0.005) (Fig. 4A). The mean frequency increased from 3.9% at week 26 to 4.6% at week 56 for the ALFQ group and from 1.88 to 3.52% for the ALFQA group (Fig. 4A). The mean frequency of LLPCs at week 26 was much lower with AH and ALFA and did not increase at week 56 (0.53 to 0.54% for AH and 0.16 to 1.14%. for ALFA). The percentage of B.63521 gp120-specific LLPCs was determined by intracellular staining with gp120-BV421 and gp120-PE (Fig. 4B). Higher percentages of these cells were induced in the bone marrow at week 56 in ALFQ and ALFQA, respectively (8.03 and 6.4%, respectively; *p* = 0.008) compared to AH and ALFA (0.88 and 2.57%, respectively) groups. Furthermore, a significant positive correlation (*r* = 0.56; *p* = 0.003) was observed between gp120-specific IgG antibody avidity score and the frequency of B.63521 gp120-specific LLPCs (Fig. 4C).

### ALFQ and ALFQA induce a higher frequency of IgG and IgA-secreting memory B cells in the bone marrow

To assess the induction of immunoglobulin-secreting memory B cells, bone marrow cells collected at weeks -4 and 48 were evaluated by ELISpot analysis following in vitro stimulation with R848 and rhIL-2 and capture of the secreted antibodies on B.63521 gp120-coated ELISpot plates. Since the number of B.63521 gp120-specific IgG-secreting cells were similar between ALFQ and ALFQA and between AH and ALFA, representative images of the ELISpot wells illustrating memory B cells secreting IgG antibodies from AH- and ALFQ-groups are shown in Fig. 4D. The number of B.63521 gp120-specific IgG and IgA-secreting B cell spot-forming units (SFU) / 10^6^ bone marrow cells are shown in Fig. 4E, F. B.63521 gp120-specific IgG SFUs were tenfold higher compared to B.63521 gp120-specific IgA-SFUs. Although, there were no significant differences between the groups, there were higher number of antigen-specific IgG (1051 vs 244) and IgA (203 vs 97) SFUs in ALFQ compared to AH group (Fig. 4E, F).

### ALFQ and ALFQA induce a significantly higher frequency of antigen-specific polyfunctional CD4^+^ and CD8^+^ T cells

**Fig. 5:**
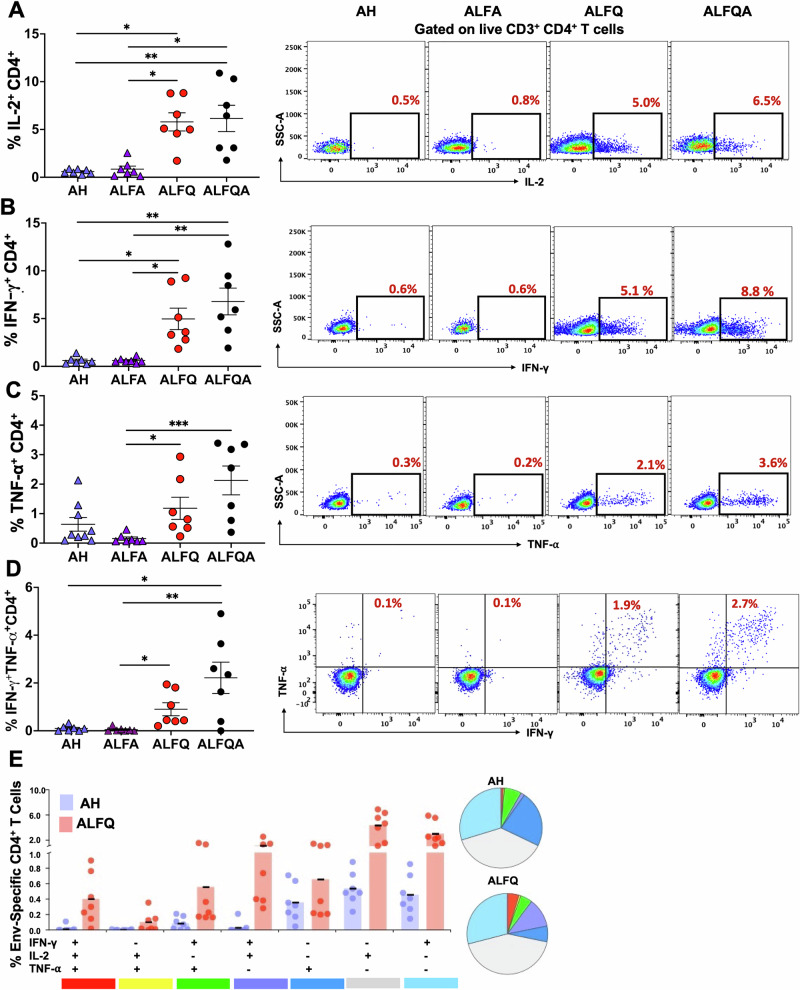
CD4^+^ T cell cytokine responses in PBMCs at week 26. **A**–**D** The percentage of HIV-1 Env-specific CD4^+^ T cells secreting **A** IL-2, **B** IFN-γ, **C** TNF-α, and **D** IFN-γ TNF-α are presented as mean ± SEM. Each individual NHP is represented as a symbol (*N* = 7/group). A representative flow plot is shown on the right. **E** Boolean gating was applied to identify polyfunctional CD4^+^ T cells. The pie chart depicts the average proportion of Env-specific CD4^+^ T cells producing all three (IFN-γ, IL-2, or TNF-α), any two, or any one cytokine. This analysis was performed using the SPICE software version 5.165. Statistical comparisons between the different adjuvant groups were performed using the Kruskal–Wallis test with Dunn’s multiple comparison test. *p < 0.05, **p < 0.009, and ***p < 0.0009. The gating strategy and the flow panels are shown in Fig. S5B and Table S9, respectively.

**Fig. 6:**
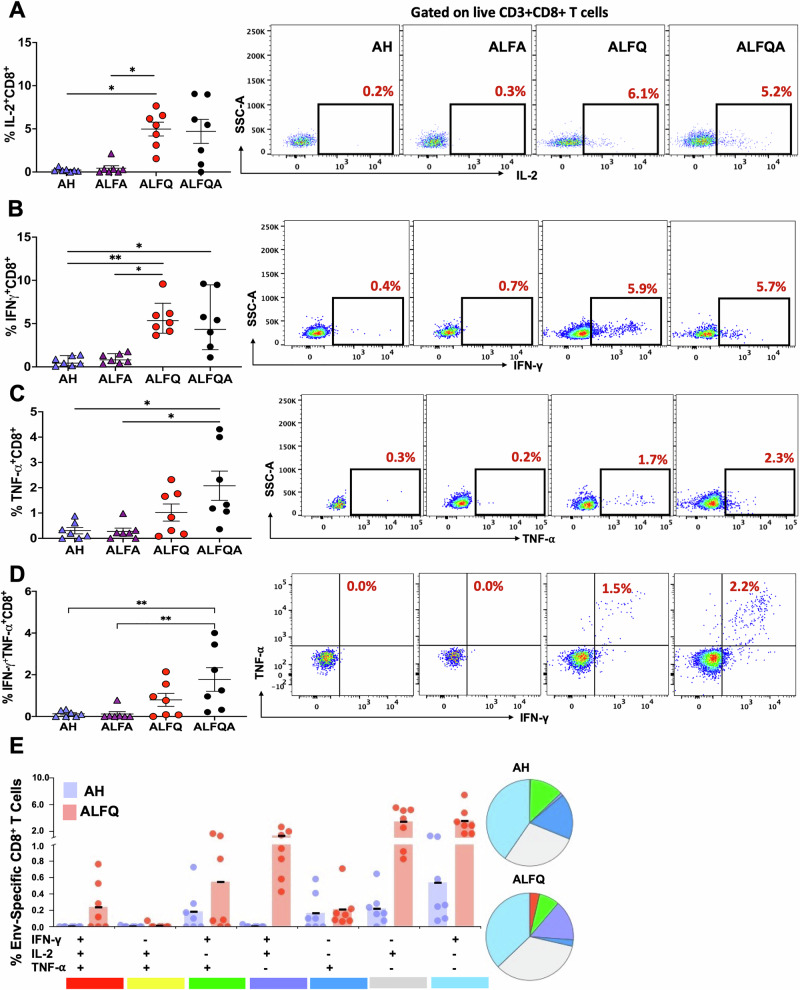
CD8^+^ T cell cytokine responses in PBMCs at week 26. **A**–**D** The percentage of HIV-1 Env-specific CD8^+^ T cells secreting **A** IL-2, **B** IFN-γ, **C** TNF-α, and **D** IFN-γ and TNF-α are presented as mean ±  SEM. Each individual NHP is represented as a symbol (*N* = 7/group). A representative flow plot is shown on the right. **E** Boolean gating was applied to identify polyfunctional CD8^+^ T cells. Pie chart and the analysis and statistical significance between the groups was performed as described above. *p < 0.01 and **p < 0.003. The gating strategy and the flow panels are shown in Fig. S5C and Table S9, respectively.

Induction of antigen-specific and polyfunctional cytokine T cell responses are important hallmarks for immunogenicity, and a strong correlation with vaccine efficacy has been demonstrated^14^. To determine if these adjuvant formulations generated antigen-specific CD4^+^ and CD8^+^ T cell cytokine responses, we assessed their frequency at week 26. PBMCs were stimulated with the gp120 subtype B peptide pool, and the intracellular cytokines were measured by intracellular cytokine staining (ICS). The flow panel and the gating strategy are shown in Table S9 and Fig. S5B, respectively. The right panels in Figs. 5, 6 show the representative flow plots. ALFQ and ALFQA vaccines induced a markedly higher percentage of IL-2 secreting (Fig. 5A, ALFQ vs AH: *p* = 0.011; ALFQ vs ALFA: *p* = 0.015; ALFQA vs AH: *p* = 0.008; ALFQA vs ALFA: *p* = 0.011), IFN-γ secreting (Fig. 5B, ALFQ vs AH: *p* = 0.015; or ALFA: *p* = 0.04; ALFQA vs AH: *p* = 0.002; or ALFA: *p* = 0.005) and TNF-α secreting CD4^+^ T cells (Fig. 5C, ALFQ vs ALFA: *p* = 0.026; ALFQA vs ALFA: *p* = 0.0009), compared to AH and ALFA vaccines. There were minimal responses with AH and ALFA for Env-specific CD4^+^ T cells co-expressing IFN-γ and TNF-α (Fig. 5D, ALFQ vs ALFA: p = 0.026; ALFQA vs AH: *p* = 0.047; ALFQA vs ALFA: *p* = 0.009) or IL-2, IFN-γ and TNF-α (Fig. 5E) compared to the polyfunctional responses induced by ALFQ and ALFQA vaccines. Similarly, ALFQ and ALFQA vaccines induced a markedly higher percentage of IL-2 secreting (Fig. 6A, ALFQ vs AH: *p* = 0.013; ALFQ vs ALFA: *p* = 0.024; ALFQA vs AH: *p* = ns; ALFQA vs ALFA: *p* = ns), IFN-γ secreting (Fig. 6B, ALFQ vs AH: *p* = 0.003; ALFQ vs ALFA: *p* = 0.022; ALFQA vs AH: *p* = 0.010; ALFQA vs ALFA: *p* = ns), and TNF-α secreting CD8^+^ T cells (Fig. 6C, ALFQA vs AH: *p* = 0.018; ALFQA vs ALFA: *p* = 0.016), compared to AH and ALFA vaccines. As was seen with CD4^+^ T cells, there were minimal responses with AH and ALFA vaccines for Env-specific CD8^+^ T cells co-expressing IFN-γ and TNF-α (Fig. 6D) or IL-2, IFN-γ and TNF-α (Fig. 6E) compared to the polyfunctional responses induced by ALFQ and ALFQA vaccines. These data demonstrate the differences in the landscape of T cell responses induced by the different vaccine-adjuvant formulations, with ALFQ and ALFQA vaccines inducing higher polyfunctional T cells.

### ALFQ and ALFQA induce higher IL-21-producing T follicular helper (Tfh) cells and higher levels of cytokines and chemokines in the serum and cell supernatants

**Fig. 7:**
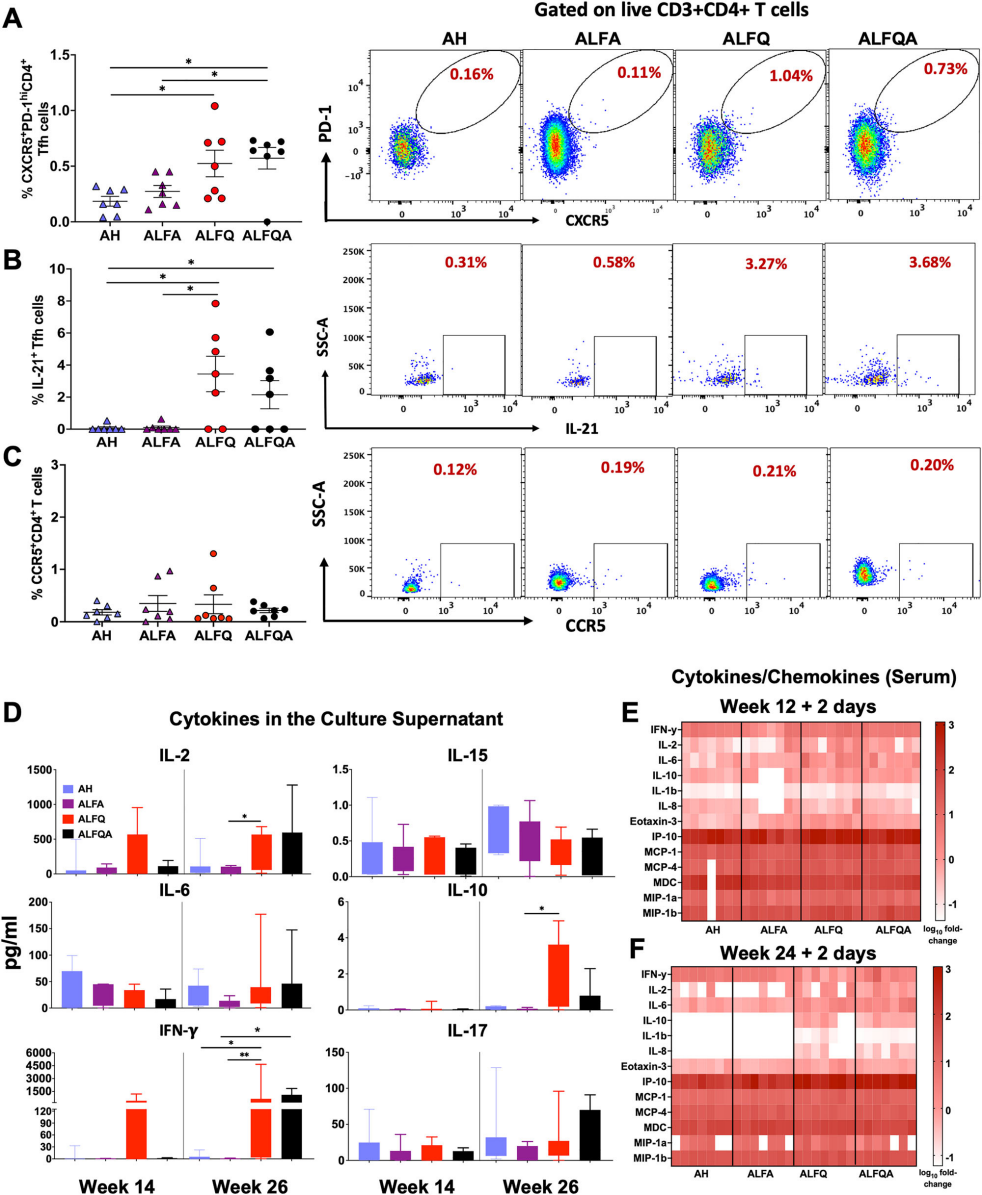
CD4^+^ T follicular helper cell (Tfh) responses in PBMCs and cytokine responses. The frequencies of **A** Env-specific Tfh cells (CXCR5^+^ PD-1^hi^ CD4^+^ T cells), **B** Env-specific IL-21-secreting Tfh, and **C** percentage of CCR5^+^ CD4^+^ T cells at week 26 were determined by flow cytometry and the data are presented as mean ± SEM. Each individual NHP is represented as a symbol (*N* = 7/group). A representative flow plot is shown on the right. **D** Cytokines in culture supernatants of PBMCs at weeks 14 and 26 were analyzed by MSD. The data from each group is shown as Box and Whisker plots. **E**, **F** Heat map of cytokines and chemokines in the sera of individual animals 2 days post-first and second (weeks 12 and 24) protein vaccinations. Statistical significance between the groups was determined using a non-parametric Mann–Whitney two-tailed test. *p < 0.02. The flow panel and the gating strategy are provided in Fig. S5C and Table S9, respectively.

Tfh cells interact with B cells and are essential for the generation of plasmablasts, memory B cells and LLPCs secreting high-affinity antibodies. Since the peak antibody responses were observed at week 26, we analyzed the PBMCs for the presence of antigen-specific Tfh cells secreting IL-21 at this timepoint. The flow panel and the gating strategy are shown in Table S9 and Fig. S5C, respectively. The right panels show the representative flow plots (Fig. 7A–C). At week 26, both ALFQ and ALFQA induced a higher percentage of CXCR5^+^PD-1^hi^ CD4^+^ Tfh cells compared to AH (*p* = 0.04 and *p* = 0.02, respectively) and ALFA vaccines (Fig. 7A). When the PBMCs were stimulated in vitro with a cocktail of subtype B Env peptides, ALFQ and ALFQA induced a higher percentage of IL-21^+^ secreting Tfh cells (Fig. 7B, ALFQ vs AH: *p* = 0.021; ALFQ vs ALFA: *p* = 0.04; ALFQA vs AH: *p* = 0.07). No significant differences were observed in the frequency of CCR5^+^CD4^+^ T cells in all groups (Fig. 7C).

PBMCs from weeks 14 and 26 were stimulated with subtype B Env peptide pools, and the supernatants were analyzed for the presence of cytokines by Meso Scale Discovery (MSD) technology. Higher amounts of IL-2 and IFN-γ were induced with ALFQ at week 14 (Fig. 7D). Following the protein boost (week 26), ALFQA induced equivalent amounts of IL-2 and IFN-γ as ALFQ, and these were significantly higher than AH and ALFA (ALFQ vs AH: *p* = 0.02; ALFQ vs ALFA: *p* = 0.005; ALFQA vs ALFA *p* = 0.04). Notably, significantly higher amounts of IL-10 (*p* < 0.03) were induced with ALFQ (ALFQ vs ALFA: *p* = 0.02; ALFQ vs AH: *p* = 0.053) at week 26. Levels of IL-6, IL-15, and IL-17 were comparable between the groups at weeks 14 and 26 (Fig. 7D).

Serum from each animal was analyzed by MSD for the presence of cytokines and chemokines 2 days post-first and second protein vaccinations. Heat map analysis showed increased levels of IL-2, IL-10, IL-8, IL-1β, and MIP-1α with ALFQ and ALFQA groups compared to AH and ALFA groups. These differences in cytokine and chemokine levels were more pronounced 2 days post-second protein vaccination (Fig. 7E, F). While the culture supernatants from PBMCs from AH and ALFA groups did not exhibit IL-10 or IFN-γ (Fig. 7D), IL-10 was present in very low amounts or absent in the sera of three out of the seven NHPs (Fig. 7E) vaccinated with ALFA-vaccine formulation. Two days post-second protein boost, there was a distinct absence of IL-10, IL-1β, and IL-8 in the AH and ALFA-vaccinated NHPs, whereas the ALFQ and ALFQA vaccinated NHPs showed low levels of IL-10 and IL-8 (Fig. 7F).

### ALFQ and ALFQA upregulate interferon signaling genes

**Fig. 8:**
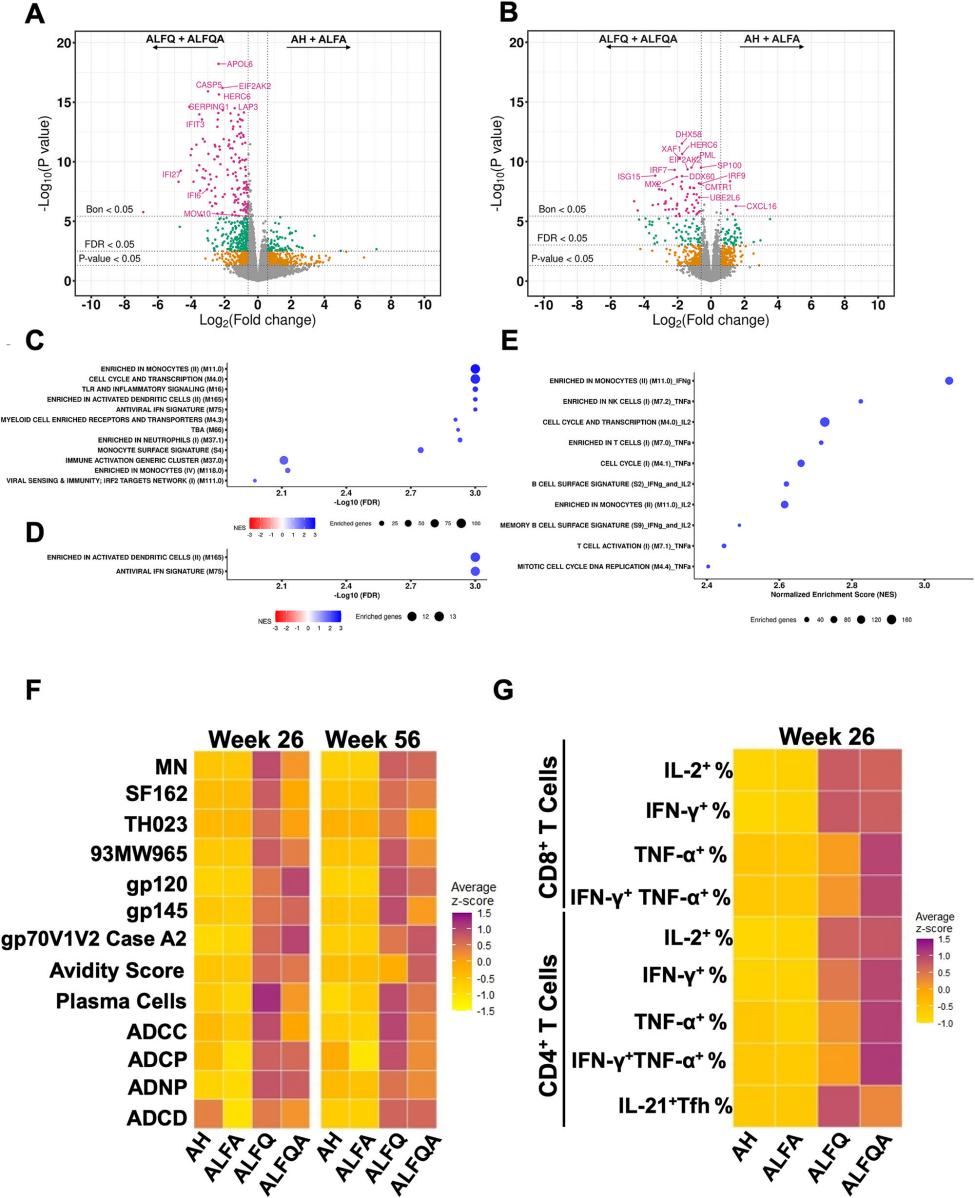
Total transcriptomics by RNA-sequencing. **A**, **B** DEG comparing the combined ALFQ/ALFQA versus AH/ALFA arms after week 12 + day 2 and week 24 + day 2, respectively. Bonferroni significant genes are shown in pink, FDR (false discovery rate) significant genes in green, nominally significant *p* value genes in orange. Bonferroni significantly interferon-stimulated genes (ISGs) are labeled in both plots. Gene set enrichment analysis (GSEA) of significant pathway enrichments for week 12 + day 2 (**C**) and study week 24 + day 2 (**D**). Normalized Expression Scores demonstrate that all identified pathways are enriched in the adjuvanted ALFQ/ALFQA arms. **E** Bubble plot showing the top ten Blood Transcription Modules (BTM) signatures associating with Env-specific CD8^+^ T cell responses in the combined ALFQ + ALFQA groups. The signature suffixes indicate the CD8^+^ T cell response for which they were enriched. The bubble size indicates the number of enriched genes per signature. The bubble color indicates the direction of the enrichment score (NES), which are all positive here. Heat maps were constructed for the normalized measures of each of the two datasets (humoral and cellular immune responses) across the respective timepoints. Heat maps of **F** humoral and **G** cellular assays at weeks 26 and 56 for humoral assays and week 26 for cellular assays. To normalize values, each Z-score for a given assay/timepoint scenario was calculated from a total pool of 28 observations (one per each NHP). The heat map color scheme yellow-orange-purple (low to high) represents a continuous scale of change. Heat maps were constructed using R statistical software v4.4.1 (R package *ggplot2*).

To evaluate the effect of the different adjuvants on gene expression, bulk RNA-sequencing was performed with PMBCs from all NHPs obtained 2 days after each of the four vaccinations. Principal component analyses by timepoints (Fig. S6A) and adjuvants (Fig. S6B–D) showed no clear patterns, other than at the last timepoint (week 24 + day 2) after vaccination (Fig. S6D). Analysis comparing results from animals vaccinated with AH vs ALFA and ALFQ vs ALFQA-vaccine formulations at both timepoints identified six differentially expressed genes (DEG) (FDR <0.05 and absolute (FC) >1.5) in the four comparisons (Table S10). These comparisons support findings from other immune responses showing equivalent responses between ALFQ and ALFQA and AH and ALFA. We therefore combined the data from NHPs vaccinated with AH and ALFA and the data from ALFQ and ALFQA to determine the impact of including QS-21. At week 12 + day 2, 431 DEG (FDR <0.05, abs (FC) >1.5) were identified with 357 genes upregulated in the ALFQ + ALFQA group and 74 genes were upregulated in the AH + ALFA group (Fig. 8A). At week 24 + day 2, a total of 170 genes were differentially expressed, with 116 and 54 genes upregulated in the ALFQ + ALFQA and AH + ALFA groups, respectively (Fig. 8B). Using gene set enrichment analysis (GSEA)^35^, we identified 12 significantly enriched pathways (NES ≥1.4 and FDR <0.01) in the ALFQ + ALFQA arm at week 12 + day 2 (Fig. 8C). After the protein boost at week 24 + day 2, we saw a consolidation of the differential transcriptome response and only two of the previously enriched gene sets were identified, namely genes enriched in activated dendritic cells (M165) and antiviral interferon signature (M75) (Fig. 8D). In contrast, no pathways were identified as being enriched in the AH + ALFA arm.

We next examined the DEGs identified with the combined ALFQ + ALFQA vs combined AH + ALFA comparisons (FDR <0.05, abs (FC) > 1.5) at the two timepoints for protein–protein interaction analysis. After the first protein-adjuvant vaccination (Week 12 + day 2), the most connected network identified by molecular complex detection (MCODE1) was related to interferon signaling and defense response to virus (Fig. S6E). When looking at the direction of the fold changes for the genes in this network, these genes were upregulated in the ALFQ/ALFQA arm, indicating a more robust interferon response. Genes in the MCODE5, 6, and 7 clusters were also all upregulated in the ALFQ/ALFQA arm, including a network related to ISG15-mediated antiviral response. MCODE2, 3, and 4 showed a mixed response, though the majority of genes in these clusters were upregulated in the ALFQ/ALFQA arm (Fig. S6E and Table S11). As with the GSEA results, two of the earlier identified networks were retained at week 24 + day 2, relating to interferon signaling (MCODE1) and ISG15-mediated antiviral responses (MCODE3), both upregulated in the ALFQ/ALFQA arm (Fig. S6F). A third network related to chemokine signaling (MCODE2) was also seen at this later timepoint. Directionality of the gene expression fold change showed most of these genes upregulated in the ALFQ/ALFQA arm, suggesting a distinct immunogenicity pathway for the QS-21 adjuvanted vaccines (Fig. S6E, F and Tables S11–13). These results point to enhanced biological perturbations observed in NHPs vaccinated with QS-21-containing adjuvant formulations.

Since the QS-21-containing adjuvant formulations induced extremely strong antigen-specific CD8^+^ T cell responses, we performed pathway enrichment (GSEA) using the frequency of Env-specific CD8^+^ T cells quantified for five immune responses (IL-2, IFN-γ, TNF-α, IFN-γ + TNF-α, and IFN-γ + IL-2) in a continuous analysis for the QS-21 saponin adjuvant groups (ALFQ + ALFQA). Signatures to interpret blood transcriptomics data were used^36^, and significantly enriched signatures associating positively with higher frequency of CD8^+^ T cells for each response within the QS-21 saponin groups (ALFQ + ALFQA) were identified using normalized enrichment scores (NES) of 1.4 or higher and Bonferroni-adjusted *p* values of less than 0.001 (Table S14). The top ten signatures associated with the frequency of Env-specific CD8^+^ T cells secreting different cytokines in the combined ALFQ group are shown as a bubble plot in Fig. 8E and Table S15. The strongest pathway associated with the frequency of CD8^+^ T cells secreting IFN-γ was a monocyte signature (NES = 3.07, *p* < 0.001).

## Discussion

Adjuvants are an important constituent of vaccines that enhance the quality and durability of vaccine-induced immune responses. Traditionally, aluminum salts have been widely used as adjuvants since they have a proven safety track record and are inexpensive. However, it is being increasingly realized that aluminum salts are not universally effective with all vaccines. Several new adjuvants are being evaluated in nonhuman primate studies and human clinical trials to address this issue^20,21,37–39^.

In this study, we compared the immunogenicity in NHPs of three Army Liposome Formulations containing either two immunostimulants, 3D-PHAD™ and AH (ALFA) or 3D-PHAD™ and QS-21 (ALFQ) or three immunostimulants, 3D-PHAD™, AH, and QS-21 (ALFQA), by mixing each one separately with an HIV-1 subtype B Env gp120 protein, B.63521. The vaccines were administered mimicking the vaccine regimen of the RV144 phase 3 trial. None of the animals exhibited any vaccine-related adverse events either locally or systemically. Analysis of the immunological responses induced by the vaccine formulations demonstrated that ALFQ and ALFQA vaccines induced the most potent and sustained immune responses. This was evident in the magnitude, durability, a slower decay rate, avidity maturation of the antibodies, higher frequency of circulating antigen-specific IL-21 secreting Tfh cells, significantly higher frequencies of persistent antigen-specific LLPCs in the bone marrow, which were seen 8 months after the last protein boost and antigen-specific polyfunctional CD4^+^ and CD8^+^ T cells compared to non-QS-21-vaccines. Higher numbers of antigen-specific IgG and IgA-secreting B cells following stimulation were also observed in the bone marrow samples. Our data is further supported by a strong positive correlation between antibody avidity and binding antibody titers, the frequency of B.63521 gp120-specific LLPCs in the bone marrow, and neutralizing antibodies. Furthermore, compared to the neutralizing antibody titers induced by AH and ALFA vaccines, ALFQ and ALFQA vaccines induced 10-fold higher titers against the pseudoviruses tested except against the homologous transmitted/founder strain B.63521 which is a more resistant isolate as compared to the other tier 1 pseudoviruses. Collectively, these factors could contribute to the durable immune responses observed with QS-21-containing vaccines.

Although, the HIV vaccine field has made the induction of durable and broadly neutralizing antibodies of greater magnitude a priority in HIV vaccine design, there is mounting evidence that in addition to antibodies mediating neutralization, non-neutralizing antibodies mediate other immunological functions through binding to Fc receptors and complement^40^. In multiple NHP studies and HIV-1 clinical trials, including studies that utilized the ALVAC/protein regimen as in the current study, vaccine-induced gene expression signatures for Fc effector functions and ADCC and ADCP responses were shown to correlate with protection^3,41–45^. In our study, ALFQ and ALFQA vaccines induced high levels of antigen-specific IgG1 and IgG2 antibodies, which have been associated with Fc effector functions. ALFQ and ALFQA vaccines induced strong ADCC, ADCP, and ADCD responses, which persisted 8 months after the last protein boost. The ADNP responses did not persist until week 56. In contrast, the ALFA vaccine was linked to the lowest level of responses. We speculate that the presence of increased numbers of recall antigen-specific-IgG and IgA-secreting cells in the bone marrow could work collaboratively to drive both ADCP and ADNP responses thereby enhancing innate immune responses. There is precedence for association with IgA or IgG and phagocytic functions mediated by monocytes or neutrophils, which in turn were associated with reduced risk of infection across vaccine regimens and challenge viruses in nonhuman primates^46^.

Since the QS-21-containing vaccines are very potent, one question would be whether they could also increase the number of CD4^+^ T cells. An increase in total or vaccine-specific CD4^+^ T cells have been shown to enhance simian immunodeficiency virus or simian-human immunodeficiency virus in rhesus macaques^47^. It has been postulated that the increased risk of HIV-1 infection among adenovirus-5 seropositive individuals in the STEP trial was due to the increased frequencies of activated adenovirus-specific CD4^+^ T cells in the mucosa^48^. In our study, at peak immunogenicity, there were no significant differences in the percentage of total CD4^+^ and CCR5^+^ CD4^+^ T cells with any of the vaccines, thus supporting our hypothesis that QS-21-containing vaccines do not increase target cells for HIV-1 infection.

Generally, protein antigens do not induce potent CD8^+^ T cell responses. In this study, the saponin-containing adjuvants induced a higher magnitude of antigen-specific CD8^+^ T cell responses. It is possible that the viral vector ALVAC, along with the saponin-containing vaccines, facilitated better cross-presentation of the antigens via MHC class I and thus enhanced the CD8^+^ T cell responses. Additionally, QS-21, when formulated in cholesterol-containing liposomes, has been shown to enhance the uptake of antigen in human dendritic cells through a receptor-independent, cholesterol-dependent mechanism, including the upregulation of the production of IL-6 and TNF-α^49^. A study by Shane Crotty et al^50^, showed induction of robust CD8^+^ T cell responses in humans with a SARS CoV-2 protein vaccine formulated with Matrix-M adjuvant which contains QS-21 (NVX-CoV2373). Matrix-M has been reported to destabilize the endosomal/lysosomal membrane and facilitate the entry of the antigen into the cytosolic compartment for MHC Class I processing and presentation^26^. Similarly, cancer vaccines adjuvanted with ISCOMATRIX, which contains Quill A (a mixture of QS saponins) as the adjuvant, elicit high frequencies of long-lasting CD8^+^ T cells in humans and animal models^51^. Thus, there is precedence that protein antigens adjuvanted with QS-21 containing saponins in the absence of a viral vector can induce strong CD8^+^ T cell responses. Nonetheless, the mechanism of ALVAC to enhance CD8^+^ T cell responses warrants further investigation in separate studies.

Different cytokine profiles can influence the quality of T cell help provided to B cells. Antigen-specific polyfunctional CD4^+^ and CD8^+^ T cell responses were induced from culture supernatants of cells from NHPs that received ALFQ or ALFQA-vaccine. After the first protein-ALFQA vaccination, antigen-specific culture supernatants showed a classic Th17 response as seen by the absence of IL-10 and IFN-γ, along with the presence of IL-6 and IL-17. This shifted to a Th1 and Th17 response as seen by the induction of IFN-γ and IL-10 after the second protein-adjuvant boost, whereas ALFQ induced a dominant and sustained Th1 type of response. Decreased levels of IL-15 at week 26, with increased levels of IL-2 at weeks 14 and 26 in the culture supernatants from ALFQ and ALFQA groups, suggest a shift from an innate dominant response to an adaptive immune response. Both culture supernatants and sera from the ALFQ group showed a similar and sustained Th1 cytokine profile, whereas sera from the ALFQA group induced a mixed Th1 and Th17 cytokine profile.

Transcriptomics of bulk PBMCs by RNAseq identified biological perturbations between the combined ALFQ/ALFQA arms and the combined AH/ALFA arms. GSEA identified several pathways that were enriched in the QS-21 adjuvanted arms. Additional analysis of the protein–protein interaction enrichment analysis performed in Metascape identified several interconnected MCODE clusters. Three networks that stood out in the ALFQ/ALFQA arms were genes related to interferon signaling and defense response to virus, ISG15-mediated antiviral responses, monocyte, TLR, and chemokine signaling. These pathways and genes were further enhanced with a protein-adjuvant boost. CCL8 is a chemokine that binds to CCR5 with high affinity and inhibits HIV-1 replication and thus could contribute to viral defense. ISG15 is an IFN-induced gene and has been shown to play a critical role in the inhibition of HIV-1 budding and release. We identified the top ten signatures associating with the frequency of Env-specific CD8^+^T cells secreting different cytokines by pathway enrichment analysis (GSEA) for the combined ALFQ and ALFQA groups. A monocyte signature was the strongest pathway that associated with the frequency of CD8^+^ T cells secreting IFN-γ. We have previously shown that a monocyte gene signature was associated with HIV vaccine protection and ADCP in the RV144 study^45^. The use of a similar vaccination regimen in the current study, indicates that ALFQ and ALFQA could be targeting effective immune responses by signaling via myeloid cells.

There are some limitations to our study. The use of HIV-1 gp120 protein and not native-like trimers as the immunogen resulted in the neutralization of tier 1 and not tier 2 viruses. We did not collect fine needle lymph node aspirates to identify specific cell populations modulated by the different adjuvant formulations and their effects on immune responses. While we did collect rectal secretions, there were no significant differences between the various groups, although there was a trend towards higher responses with the ALFQ vaccine. The study was designed as an immunogenicity study and not as a vaccine efficacy study. Therefore, we cannot predict whether ALFQ and ALFQA-vaccines would also induce protection. Despite these limitations, our study highlights the potency of the liposomal saponin-containing adjuvants, ALFQ and ALFQA. The overall immune responses generated by ALFQ and ALFQA were superior to that seen with AH and ALFA, as reflected by the heat maps (Fig. 8F, G). The AH and ALFA-vaccine groups had lower values in all humoral and cellular immune assays conducted, compared to the ALFQ and ALFQA-vaccine groups, and this pattern was consistent across timepoints.

Inclusion of three immunostimulants did not further enhance the immune response, nor did it induce a suppressive effect. The lack of an additive effect of the adjuvant combination is striking. There was little to no improvement on the addition of liposomal MPLA to Alum (AH vs ALFA) and Alum to liposomal MPLA + QS-21 (ALFQ vs ALFQA). It is possible that if we had used lower concentrations of AH or lower concentrations of MPLA and QS-21, we might have seen an additive or synergistic effect, as the concentrations we used were likely at maximum levels for the immune responses, so the addition of a third adjuvant did not have a substantial effect. Currently, ALFQ and ALFQA are being evaluated in several HIV-1 vaccine phase 1 clinical trials with monomeric or trimeric HIV-1 envelope proteins [RV575 (NCT05423418), RV546 (NCT04658667), RV591 (NCT06205056)]. Our NHP data are encouraging and further support the inclusion of potent adjuvants, ALFQ or ALFQA, in vaccine formulations to induce holistic immune responses, including potent antigen-specific CD8^+^ T cell responses and higher expression of genes and pathways involved in innate and antiviral responses. The identification of a potent adjuvant such as ALFQ/ALFQA that can enhance CD8^+^ T cell immune responses could substantially minimize the complications of vaccination regimens involving heterologous viral vectors if the same adjuvant can balance antibody and CD8^+^ T cell responses. These data could impact and guide HIV vaccine design strategies.

## Methods

### Immunogen-adjuvant formulation

Engineering grade recombinant subtype B gp120 transmitted founder (T/F) Env protein B.63521 Δ11mutc produced by KBI Biopharma (Durham, NC) in stably transfected DG44 CHO cells and purified using column chromatography was provided by Dr. Barton Haynes (Duke University). B.63521Δ11mutC has an 11-amino acid (aa) truncation at the N terminus of gp120 that enhances antigenicity of the V2 and C1 epitopes that are targets for antibody-dependent cell-mediated cytotoxicity (ADCC)^52,53^ and prevents gp120 dimerization^53^. B.63521Δ11mutC also has a mutated V3 loop (SIR-GPGQT) to prevent clipping of gp120 when expressed in CHO cells for manufacturing^54^. HIV Env gp145 C.6980 protein was produced in CHO cells and was kindly provided by the Division of AIDS, NIAID, NIH. This protein, which was down-selected from four East African HIV-1 subtype C strains, was derived from an individual in the acute phase of HIV-1 infection. The gp145 protein assembles into physical trimers with no engineered trimerization motifs and binds many of the well-described broadly neutralizing MAbs^55^. Recombinant gp70V1V2 fusion protein purified from 293T cells specific to clade B (Case A2) was purchased from Immune Technology Corporation. The fusion protein consists of a C-terminal His-tagged gp70 from murine leukemia virus and V1V2 loops from HIV-1 Clade B/Case A2. HIV-1 96ZM651 gp120 recombinant protein was obtained through the NIH AIDS Reagent Program, NIH.

ALF containing DMPC, DMPG, cholesterol, and synthetic MPLA, 3D-PHAD™ (molar ratio of 9:1:7.5:0.36 or 9:1:12.2:0.36), the latter for vaccine preparations adjuvanted with ALFQ, were prepared by the lipid deposition method. Lipids were dried by rotary evaporation followed by overnight desiccation, rehydrated with molecular-biology grade water (Quality Biological), or with Sorensen’s PBS, pH 6.2, microfluidized, and sterile-filtered, followed by lyophilization or addition of QS-21 to the filtered ALF to form ALFQ. One hundred micrograms of B.63521 gp120 protein formulated in 20 mM sodium phosphate, 150 mM NaCl, 0.02% polysorbate 80, pH 6.5, was adsorbed to Alhydrogel® (AH) in PBS, pH 7.4, or it was then added to lyophilized ALF to generate ALFA, or the protein was mixed with ALFQ in a 1:1 volumetric ratio. Alternatively, B.63521 gp120 protein adsorbed to AH was then mixed with ALFQ to generate ALFQA. Each vaccine dose in a 500 µL volume contained 100 µg MPLA, 100 µg protein, and either 300 µg Al^3+^ (AH) or 50 µg QS-21 or both immunostimulants.

### Rhesus macaque vaccination

Twenty-eight colony-bred Indian origin male and female Rhesus macaques (*Macaca mulatta*), 50–56 months old, negative for SIV and simian T cell lymphotropic virus, were randomized into four groups (*N* = 7/group) with an even distribution of age, weight, and sex. Animals were cared for in accordance with local, state, federal, and institutional policies and were housed in the animal facility of WRAIR, which is an American Association for Accreditation of Laboratory Animal Care International (AAALAC International)-accredited facility. Studies were carried out in accordance with the recommendations in the Guide for the Care and Use of Laboratory Animals of the National Institutes of Health. The protocols were approved by the Institutional Animal Care and Use Committee at WRAIR [Assurance number D16-00596 (A4117-01)]. Animals were primed at months 0, and 1 with 1.14 × 10^7^ pfu canarypox vector ALVAC vCP2438 expressing the HIV-1 envelope glycoprotein of subtype C ZM96.C strain, along with gp41 transmembrane sequence, gag, and protease from subtype B LAI strain. The two boosting vaccinations consisted of ALVAC vCP2438 and HIV-1 Env gp120 subtype B protein, B.63521 formulated individually with each of the four different adjuvants and administered contralaterally at 100 μg/dose in 0.5 mL at months 3 and 6 following the prime. All vaccinations were intramuscular, alternating right and left quadriceps for each product with each vaccination. Animal handling was consistent throughout the study, and no animal illnesses were noted. All immunizations, blood, mucosal, and bone marrow samplings were performed under sedation with Ketamine (5–10 mg/kg) with dexmedetomidine (0.015–0.02 mg/kg) injected I.M., followed by atipamezole I.M. (0.015–0.02 mg/kg) administered immediately after the procedure to reverse the sedation. Prior to the bone marrow aspiration procedure, animals were provided Buprenorphine (0.01–0.03 mg/kg, I.M.) as an analgesic. Additionally, NHPs received carprofen (4.4 mg/kg, S.C.) for inflammation. Animals were weighed, and temperature, pulse, and respiration were measured each time the animals were sedated. At the end of the study, the animals were released back to the WRAIR nonhuman primate pool.

### Specimen collection and processing

Specimens were collected at various timepoints as shown in Fig. 1A. PBMCs were isolated from whole blood collected in BD Vacutainer CPT tubes containing 0.1 M sodium citrate, plasma was collected, residual RBCs were lysed, washed, and cryopreserved in liquid nitrogen. Serum obtained from coagulated blood was stored at −80 °C.

Bone marrow aspirates were taken from either the greater trochanter of the femur, the greater tuberosity of the humerus, or the iliac crest of the pelvis and collected into EDTA-containing tubes, passed through a 100 µm cell strainer, washed with PBS, followed by underlaying of Ficoll. The cells at the interface layer were collected, washed, RBCs lysed, and cryopreserved in liquid nitrogen.

### Calculation of antibody decay rates

The average endpoint titers for gp120 B.65321 and gp70V1V2 proteins at each timepoint were calculated as a geometric mean with corresponding upper and lower bound confidence limits around the mean, then transformed into log10 scale. Half-lives for the (non-transformed) titer decay was calculated as a function of change in titer value between weeks 26 and 48 or between weeks 26 and 56 using the exponential decay method. Tests for statistical difference between group decay rates were calculated by ANOVA. Pairwise differences between titers for each feature (respectively) were calculated with Fisher’s least square difference (LSD) method.

### B cell ELISpot analysis of bone marrow samples

ELISpot analysis was conducted as previously described with modifications^29^. Multiscreen PVDF plates (Millipore) were coated with either B.65321 gp120 protein or with anti-IgG (MT91/145) or anti-IgA (MT57) monoclonal capture antibodies. Plates were blocked with sterile assay media (RPMI with 10% FBS). Bone marrow cells from week 48 that were stimulated with R848 (1 µg/mL) and rhIL-2 (10 ng) for 50 h were added to the wells and incubated in the CO_2_ incubator at 37 °C for 17 h to allow Ig secretion. The plates were washed, biotinylated-anti-human IgG or anti-human IgA was added, followed by the addition of 100 µL of streptavidin-alkaline phosphatase (1:1000 dilution), and 100 µL of 5-bromo-4-chloro-3-indolyl phosphate/nitro blue tetrazolium substrate. Plates were rinsed gently with tap water and air-dried overnight. Counting and data analysis were conducted using the AID Autoimmun Diagnostica GmbH ELISpot reader and software. B cell responses were considered positive when the mean spot count exceeded the mean ± 3 SD of the negative control wells.

### Neutralizing antibody assessment

Neutralizing antibodies were measured as a function of reduction in luciferase (Luc) reporter gene expression after a single round of infection in TZM-bl cells as previously described^56^, with modifications for high-throughput analysis. A multi-subtype panel of pseudoviruses (PSVs) was assessed, including tier 1 subtypes B (MN.3, SF162), C (93MW965), CRF01_AE (TH023), tier 2 subtype B (WITO) and B.63521 (1012.11.TC21.3257, Transmitted/Founder Env) and murine leukemia virus (MuLV) (nonspecific control). Plasma was diluted 1:10 in growth medium and serially diluted using the Biomek NXP liquid handler (Beckman Coulter, Indianapolis, IN), transferred to 384-well culture plates, and incubated with an equal volume of PSV for 45 min at 37 °C. TZM-bl cells (3 × 10^3^ cells/well) mixed with DEAE-dextran were added to each well and incubated for an additional 48 h. Relative light units were detected with the SpectraMax Paradigm Microplate Reader (Molecular Devices, Sunnyvale, CA) using the Bright-Glo Luciferase Assay System (Promega Corporation, Madison, WI). Neutralization dose–response curves were fitted by nonlinear regression using the LabKey Server, and the final titer is reported as the reciprocal of the dilution of plasma necessary to achieve 50% neutralization (50% inhibitory dose). This assay has been formally optimized and validated^57^ and was performed in compliance with Good Clinical Laboratory Practices, including participation in a formal proficiency testing program^58^.

### Flow cytometry staining for Env-specific plasma cells in the bone marrow

B.63521 gp120 protein was separately conjugated to either BV421 or to PE using DyLight 405® conjugation kit (ab 201798) or R-PE conjugation kit (ab 102918), respectively, as per the manufacturer’s instructions. Bone marrow antigen-specific plasma cells (1 × 10^6^) were first incubated with Fc block and LIVE/DEAD Fixable Aqua Dead Cell Stain Kit (Invitrogen/Thermo Fisher Scientific, 1:1000), washed, surface stained with a cocktail of fluorescently labeled antibodies specific for plasma cell subsets (Table S8), washed, and fixed/permeabilized in the dark using the eBioscience™ Intracellular Fixation and Permeabilization Buffer Set (Thermo Fisher Scientific, 88-8824- 00) as per the manufacturer’s instructions. Cells were then incubated with gp120-BV421 and gp120-PE and intracellular antibodies (Table S8), washed with permeabilization buffer, and resuspended in FACS buffer. Appropriate single-color compensation controls and Fluorescence minus one control (FMO) were prepared simultaneously and were included in each analysis. Flow cytometric analysis was performed on a BD LSR II flow cytometer, and data were acquired using Diva software (BD Biosciences). The results were analyzed using FlowJo software version 10.7.1 (BD Biosciences). The gating strategy applied for the evaluation of flow cytometry-acquired data were provided in Fig. S5A.

### RNA isolation and transcriptomics

Transcriptomics was performed using RNAseq from peripheral blood cells as described previously^44,59^. Briefly, total RNA was extracted from 10,000–100,000 or 30,000–6.5 million cryopreserved PBMC collected before immunization and 2 days after each of the two ALVAC and two protein immunizations based on availability of samples (Table S13) using the Single Cell RNA Purification Kit (Norgen Biotek Corp) or the PureLink RNA Mini Kit (Thermo Fisher Scientific). Amplified cDNA was made from 6.66 to 10 ng of RNA using the SMART-Seq v4 Ultra Low Input RNA kit (Takara Bio Inc.), spiked with 5 µL of 1:20,000 diluted ERCC Mix 1 or 2 (Thermo Fisher Scientific). Sequence-ready libraries were generated using the Illumina DNA Library Prep Kit, quantified on a MiSeq, and sequenced on the NovaSeq 6000 (Illumina) per manufacturer’s protocol.

### Gene expression analyses

Raw sequencing data were converted to FASTQ using bcl2fastq2 v.2.20.0 (Illumina); low-quality bases were removed using Trimmomatic v0.39 and FastQC v0.11.8^60^. Reads were mapped to the Rhesus Macaque genome (Mmul_10) using HISAT2 v2.1.0^61^. Subread *featureCounts* v1.6.4 was used for gene expression quantification of protein-coding genes, and trimmed mean of *M* values (TMM)-based normalization was implemented within edgeR v3.36.0^62^. Genes aligning to highly divergent regions of the genome, deriving from mitochondria, coding for ribosomal proteins and hemoglobin-related genes were excluded from the count matrix. Only genes with counts above 10 in a minimum of 15 samples were retained, and subsequently used for differentially expressed gene (DEG) analyses. Principal component analyses (PCA) were performed to visualize gene expression data by timepoint and adjuvant.

DEGs were identified using an analytical pipeline described previously^59^. Briefly, samples belonging to the AH and ALFA adjuvant arms were grouped together and compared against those from the ALFQ and ALFQA arms, at the Week 12 + day 2 and Week 24 + day 2 timepoints. The general linear model method of “edgeR” was used to identify DEGs in the groups, which were then visualized using volcano plots (Fig. 8A, B). Normalized count matrices were also subjected to gene set enrichment analysis (GSEA) using the blood transcription modules and the GSEA software (v4.1.0)^35,36^. Significantly enriched signatures were identified using absolute normalized enrichment scores (NES) of 1.4 or higher and Benjamini–Hochberg adjusted *p* values or a false discovery rate (FDR) lower than 0.05 at the two timepoints (Fig. 8C, D). DEG (FDR <0.05, abs(FC) >1.5) were further subjected to pathway analysis using Metascape (v3.5.20230501)^63^ with express analysis as *H. sapiens*. The MCODE algorithm^64^ was used for protein–protein interaction enrichment analysis as implemented in Metascape. Proteins that did not form physical interactions with other proteins in the DEG lists were pruned, and only networks with five or more gene members were visualized (Fig. S6). Each MCODE network was analyzed independently for pathway and process enrichment. The three best-scoring terms by *p* value for each network are listed in Tables S11, S12.

#### Gene set enrichment analysis (GSEA)

GSEA was performed using the frequency of Env-specific CD8^+^ T cells quantified for five immune responses (IL-2, IFN-γ, TNF-α, IFN-γ + TNF-α, and IFN-γ + IL-2) in a continuous analysis for the QS-21 saponin adjuvant groups (ALFQ + ALFQA). Signatures in the Blood Transcription Modules (BTM)^36^ was used, and significantly enriched signatures associating positively with higher frequency of CD8^+^ T cells for each response within the QS-21 saponin groups (ALFQ + ALFQA) were identified using normalized enrichment scores (NES) of 1.4 or higher, and Benjamini–Hochberg adjusted *p* values or a false discovery rate (FDR) less than 0.01.

### Envelope-specific antibody avidity determination by surface plasmon resonance (SPR)

Antibody avidity determinations for the serum samples were conducted using the Biacore 4000 surface plasmon resonance (SPR) system as previously described^31,65,66^. The immobilizations were performed using a standard amine-coupling kit as previously described^31^. The CM5 sensor chip surface was activated with a 1:1 mixture of 0.4 M 1-ethyl-3-(3-dimethylaminopropyl) carbodiimide hydrochloride (EDC) and 0.1 M *N*-hydroxysuccinimide (NHS) (Cytiva) for 600 s. Since the density of the immobilized surface could potentially affect avidity, different immobilized surfaces of a ligand were utilized. B.63521 gp120 protein was immobilized in 10 mM sodium acetate pH 4.5 on spots 1, 2, 4, and 5 of flow cells 1–4 resulting in 2552–3660 RU using 3 μg/mL (Flow cell1); 5041–5169 RU using 5 μg/mL (Flow cell 2); 5652–5747 RU using 10 μg/mL (Flow cell 3); and 6107–6410 RU using 15 μg/mL (Flow cell 4), while spot 3 in each flow cell was left unmodified and served as the reference. The immobilized surface was then deactivated with 1.0 M ethanolamine-HCl, pH 8.5, for 600 s. Following the surface preparation, heat-inactivated serum samples were diluted 1:50 in running buffer (10 mM HEPES, 300 mM NaCl, 0.05% Tween 20, pH 7.4) and injected onto the antigen-immobilized surface for 250 s, followed by dissociation for 1800 s. To regenerate the surface, a 150 mM HCl solution was injected over the surface twice for 60 s. Four independent replicates for each measurement were collected at a rate of 10 Hz, with an analysis temperature of 25 °C. All sample injections were conducted at a flow rate of 10 μL/min. Data analysis was performed using Biacore 4000 evaluation software 4.1 with double subtractions for an unmodified surface and buffer for the blank. Fitting was conducted using the dissociation mode integrated with evaluation software 4.1. The data were shown as avidity score (RU/*K*_d_), which was determined using the ratio of binding response (response unit, RU) to dissociation rate (*K*_d_).

### Processing of rectal swabs

Rectal secretions were collected at study timepoints shown in Fig. 1A using Weck-cel and stored at −80 °C. Rectal mucosal swabs collected on day 30 (pre-vaccination), weeks 26 and 56 were thawed, assessed for stool and blood contamination, and extracted with ice-cold sterile-filtered DPBS without Ca^2^^+^ and Mg^2^^+^ containing 2.5% BSA and 2% Roche mini protease inhibitor cocktail, followed by centrifugation at 4000 rpm at 4 °C for 15 min. The supernatant was placed into chilled Costar SpinX tube containing 0.2 µm cellulose acetate filter and spun in a microcentrifuge for 20 min at 16,000 rpm. The extracted filtered rectal samples were used immediately for antibody binding analysis, and the remaining samples were aliquoted and stored frozen at −80 °C.

### Envelope-specific antibody analysis in rectal secretions by surface plasmon resonance

Antibody binding determinations were conducted using a Biacore 4000 surface plasmon resonance (SPR) system. The immobilizations were performed in a solution containing 10 mM HEPES and 150 mM NaCl (pH 7.4) using a standard amine-coupling kit, as previously described^31^. The CM5-S series chip surface was activated with a 1:1 mixture of 0.4 M 1-ethyl-3-(3-dimethylaminopropyl)carbodiimide hydrochloride (EDC) and 0.1 M *N*-hydroxysuccinimide (NHS) for 600 s (Cytiva). B.65321 gp120 protein or anti-monkey IgG were coupled for 720 s on the chip. The immobilized surface was then deactivated with 1.0 M ethanolamine-HCl (pH 8.5) for 600 s. Following surface deactivation, 12,000 RU of B.63521 and 24,000 RU of anti-IgG were captured on the chip. Following surface preparation, extracted rectal secretions diluted 1:2, 1:5, and 1:10 in running buffer (10 mM HEPES, 300 mM NaCl, and 0.05% Tween 20, pH 7.4) or monkey IgG (1.22 ng/mL–20 µg/mL) were injected onto the anti-IgG surface with replicate spots for 320 s, followed by a 1800 s dissociation period. The bound surface was then enhanced with a 240 s injection of 30 µg/mL of the secondary antibody, goat anti-monkey IgG. For each sample or controls, four replicates for each dilution were collected at a rate of 10 Hz, with an analysis temperature of 25 °C. To regenerate the bound surface, 175 mM HCl was injected over the surface for 70 s. The concentration of Env-specific IgG antibodies in the rectal secretions was determined using the standard curve. Fitting was conducted using the dissociation mode integrated with Evaluation software 4.1 with double subtractions (unmodified surface and buffer for the blank). The data were normalized and reported as specific IgG (pg)/ total IgG (ng).

### Analysis of envelope-specific IgG binding antibody responses by ELISA

Serum IgG titers against B.63521 gp120, gp145 acute subtype C, gp70V1V2 Case A2 (subtype B), 96ZM651 gp120 proteins, cyclic V1 and V2 peptides derived from B.63521 sequence was determined by an ELISA as previously described^67,68^. Briefly, each well of a 96-well Immulon 2 “U” bottom plate was coated with 100 µL of either gp120 B.63521 or gp145 acute subtype C or 96ZM651 gp120 (subtype C) protein or gp70V1V2 Case A2 proteins (1 µg/mL) in Dulbecco’s PBS, pH 7.4, and placed for 2 h at 37 °C. Following blocking with buffer (blocking buffer PBS containing 0.5% Casein and 0.5% BSA, pH 7.4), the plates were stored overnight at 4 °C. The next day, the blocking buffer was dumped, and individual serum samples were serially diluted twofold in blocking buffer and added to triplicate wells. The plates were incubated at RT for 1 h, washed in PBS buffer, pH 7.4, containing 0.1% Tween 20, followed by the addition of secondary antibody specific to IgG (Horseradish peroxidase (HRP)-conjugated goat anti-monkey IgG, gamma chain specific; Alpha Diagnostic International) for 1 h at RT. Color was developed by the addition of 2,20-Azinobis [3-ethylbenzothiazoline-6-sulfonic acid]-diammonium salt (ABTS) HRP substrate (KPL/Seracare) for 1 h at RT. The reaction was stopped by the addition of 100 µL of 1% SDS per well and the absorbance (A) was measured at 405 nm (A405) using a Spectramax Plus plate reader (Molecular Devices, San Jose, CA) within 30 min of stopping the reaction. Matched pre-immune serum was used as the negative control for each animal. Sera from NHPs immunized with gp145 protein and ALFA from a different study^68^ served as the positive control and were included on each plate. The results are expressed as endpoint titers, defined as the reciprocal dilution that gives an absorbance value that is greater than or equal to twice the background value (antigen-coated wells that did not contain the test sera, but had all other components added). For the peptide ELISA, wells were coated overnight with 0.2 μg/well of Streptavidin followed by the addition of 0.1 μg/well of biotinylated cyclic-V1 or -V2 peptides (JPT Peptide Technologies) in bicarbonate buffer, pH 9.6, for 1 h at 37 °C. The wells were blocked overnight with 0.5% milk, 0.1% Tween 20 in phosphate-buffered saline (PBS), pH 7.4 at 4 °C. The rest of the procedure was identical to the protein ELISA described above.

### Analysis of envelope-specific IgG subclass antibodies by ELISA

Each well of a 96-well Immulon 2 “U” bottom plate was coated overnight at 4 °C with 100 µL of 1 µg/mL of B.63521 gp120 in PBS or eleven rows were coated in triplicate with twofold serial dilutions of rhesus IgG1 or IgG2 (Nonhuman Primate Reagent Program, PR-2408 and PR-2409) starting at 1 µg/mL in PBS to generate a standard curve. The plates were incubated overnight at 4 °C. Plates were washed with PBS containing 0.05% Tween 20, pH 7.4, and blocked for 30 min at RT with reagent buffer (0.1% bovine serum albumin in wash buffer). Twofold dilutions of serum in reagent buffer were then added to the wells coated with gp120. Reagent buffer was added to the wells coated with the standard. Following overnight storage at 4 °C, the plate was washed and reacted for 1 h at 37 °C with 1 µg/mL of the relevant monoclonal antibody from the Nonhuman Primate Reagent Resource: mouse anti-rhesus IgG1 (PR-1234) or IgG2 (PR-0004) conjugated to HRP. The plates were washed, and 1-Step Ultra TMB ELISA substrate solution (Fisher Scientific) was added, and the plates were incubated for 15 min at RT. Color development was stopped. by the addition of 100 µL of 2 M sulfuric acid (Fisher Scientific). The absorbance was recorded at 450 nm on a Spectramax Plus plate reader (Molecular Devices, San Jose, CA). SoftMax Pro software (Molecular Devices) and GraphPad Prism 9.0 was used to construct a standard curve and determine concentrations of the antibody.

### Analysis of cytokines in the serum and cellular supernatants by MSD

Cytokines were measured in the sera of vaccinated animals 2 days following the first and second protein vaccinations, respectively (study week 12 + day 2; study week 24 + day 2). Cryopreserved PBMCs (study week 26) were thawed and rested overnight at 37 °C, 5% CO_2_. They were then stimulated with 2 µg/mL consensus subtype B envelope peptide pool (NIH AIDS Repository) for 6 h and then the supernatants were collected and analyzed using Meso Scale Discovery (MSD) custom plates containing NHP-specific-proinflammatory, anti-inflammatory cytokines, and chemokines. The assay was performed according to the manufacturer’s protocol.

### Generation of NK92.rhCD16 cell line

NK92 cells (ATCC CRL-2407) are a human NK-cell line derived from an individual with malignant non-Hodgkin lymphoma. The cells have natural cytotoxicity activity but lack expression of FcγRIIIa (CD16). We produced NK92 cells that stably express rhesus macaque FcγRIIIa using the Amphotropic Platinum Retrovirus Expression System (Cell Biolabs, San Diego, CA). Briefly, the extracellular domain of the rhesus macaque *FCGR3A* gene (GenBank JQ038005.1) was synthesized as a chimera with the transmembrane and intracellular signaling domain of human FcεRIγ, similar to that previously described^69^. This construct was subcloned into plasmid pMXs-Puro retroviral expression vector using BamHI and NotI sites and transfected into the Plat-A retrovirus packaging cell line using FuGENE (Promega, Madison, WI). Supernatant containing retrovirus was used to transduce NK92 cells, and flow cytometry cell sorting with anti-CD16 antibody (clone 3G8, BD Biosciences, San Jose, CA) was used to select cells that expressed the receptor on the cell surface. A clonal population was isolated by expansion after single-cell dilution, the sequence of the inserted gene was confirmed by targeted PCR amplification, and Sanger sequencing (Genewiz, Morrisville, NC) and the stability of cell-surface expression of rhCD16 was confirmed by regular monitoring by flow cytometry.

### Antibody-dependent cellular cytotoxicity (ADCC) assay using B.63521 gp120-coated target cells

ADCC activity was assessed as previously described using EGFP-CEM-NKr-CCR5-SNAP cells that constitutively express GFP as targets^70^. Briefly, B.63521 gp120 protein-coated target cells were washed and labeled with SNAP-Surface® Alexa Fluor® 647 (New England Biolabs) as recommended by the manufacturer. Heat inactivated, serially diluted (7 tenfold dilutions starting at 1:10) plasma samples (100 μL) from weeks 0, 14, 26, and 56, were added to wells of a 96-well V-bottom plate (MilliporeSigma). Target cells (5000 in 50 μL) and 250,000 human PBMCs (50 μL) as effectors were added to each well to give an effector/target (E/T) ratio of 50:1. The plate was incubated at 37 °C for 2 h, washed, and the cells were resuspended in 200 μL of a 2% PBS–paraformaldehyde solution and acquired on a BD LSR II equipped with a high-throughput system (BD Biosciences, San Jose, CA, USA). Specific killing was measured by the loss of GFP from SNAP-Alexa647^+^ target cells. Target and effector cells cultured in the presence of R10 media were used as background. HG107 monoclonal antibody (NIH AIDS reagent program), was used as a positive control. Normalized percent killing was calculated as: (killing in the presence of plasma − background)/(killing in the presence of HG107 − background) × 100. ADCC endpoint titer is defined as the reciprocal dilution at which the percent ADCC killing was greater than the mean percent killing of the background wells containing medium, target and effector cells, plus three standard deviations.

### Antibody-dependent cellular cytotoxicity (ADCC) flow-based GTL assay

Heat-inactivated plasma samples from weeks 0, 14, 26, and 56 were used in the flow-based GTL ADCC assay^71^. Target cells (CEM.NKR cells expressing the CCR5 co-receptor) were coated with B.63521 gp120 (cognate antigen) or MN or TV1 gp120 proteins (surrogate of recombinant ALVAC). The cut-off for positivity in the GTL assay was >5% of Granzyme B (GzB) activity in the target cells after subtracting the background represented by the condition where target and effector cells were incubated in the absence of antibodies. The percentage of NK-killed gp120-coated targets, reported as % singlets, was evaluated using area scaling analysis of the GzB+ cells observed at the dilution of the peak activity according to the published procedure^72^.

### Luciferase-based (Luc) ADCC assay

Heat-inactivated plasma samples were tested using infected target cells in the Luc ADCC assay against the IMCs^73^. These included subtype C 96ZM651 that matched the ALVAC recombinant construct used in the vaccine regimen; WITO, a representative subtype B to reflect the boost gp120, and TV1, a representative subtype C gp120. The analysis of the results was conducted after subtracting the background detected with the pre-immunization samples. After background subtraction, results were considered positive if the % specific killing was above 10%. The effector cells used in GTL and Luc assays were an NK-cell line (NK92) expressing Rhesus macaque CD16 receptor, generated by Dr. Pollara.

A magnitude-breadth (M-B) curve was used to plot the ADCC response (either Titer or Percent Peak Activity) and breadth (number of isolates targeted) of an individual sample assayed against a panel of three different HIV-1 IMCs for the Luc assay and three different gp120s for the GTL assay. The AUC of an M-B curve provides an overall summary of the M-B profile and equals the average ADCC response over the three targets tested for both Luc and GTL Assays.

### Antibody-dependent cellular phagocytosis (ADCP)

ADCP was measured as previously described^74^ using biotinylated B.63521 gp120 protein at a biotin to protein ratio of 1:50 with 100 μL of 300 or 1000-fold diluted plasma before addition of THP-1 cells (20,000 cells per well; MilliporeSigma, Burlington, MA, USA). Fluorescence of fixed cells was evaluated on an LSR II flow cytometer (BD Bioscience). The phagocytic score was calculated by multiplying the percentage of bead-positive cells by the geometric mean fluorescence intensity (MFI) of the bead-positive cells and dividing by 10^4^.

### Antibody-dependent neutrophil phagocytosis (ADNP)

Biotinylated B.63521 gp120 protein-yellow-green neutravidin-fluorescent beads were prepared as described above and incubated with 100 μL of 300-fold diluted plasma, followed by the addition of ACK-lysed fresh human peripheral blood leukocytes as the effector cells. After incubation at 37 ^o^C for 1 h, the cells were washed, surface-stained, fixed with 4% formaldehyde solution (Tousimis, Rockville, MD, USA), and evaluated on a LSR II flow cytometer (BD Bioscience). Antibodies used for flow cytometry were anti-human CD3 AF700 (clone UCHT1, BD Biosciences), anti-human CD14 APC-Cy7 (clone MϕP9; BD Biosciences), and anti-human CD66b Pacific Blue (clone G10F5, Biolegend). The phagocytic score was calculated by multiplying the percentage of bead-positive neutrophils (SSC high, CD3^−^ CD14^−^ CD66^+^) by the geometric MFI of the bead-positive cells and dividing by 10^4^.

### Antibody-dependent complement deposition (ADCD)

The ADCD assay was adapted from Fischinger et al.^75^. Briefly, biotinylated B.63521 gp120 protein-red neutravidin-fluorescent beads labeled as described above were incubated with diluted plasma samples before addition of THP-1 cells (MilliporeSigma, Burlington, Massachusetts, USA). Antibody coated beads were washed and resuspended in 200 μL of guinea pig complement (CL4051, Cedarlane, Burlington, Canada), which was prepared at a 1:50 dilution in Gelatin Veronal Buffer with Ca^2+^ and Mg^2+^ (IBB-300x, Boston BioProducts, Ashland, MA), incubated at 37 ^o^C for 20 min, washed in PBS containing 15 mM EDTA, and stained with an anti-guinea pig complement C3 FITC (polyclonal, Thermo Fisher Scientific). Fluorescence was evaluated on an LSR II flow cytometer (BD Bioscience).

### Flow cytometry phenotyping and intracellular cytokine staining (ICS)

Cryopreserved PBMCs were quickly thawed in a 37 °C water bath and then transferred to pre-warmed R10 [RPMI 1640, 10% FBS, 2 mM l-glutamine, 100 U/mL penicillin G, 100 μg/mL streptomycin] followed by washing. Cells were then resuspended at 1–2 million cells/mL in R10 in 15 mL tubes and rested overnight at 37 °C, 5% CO_2_. After overnight rest, cells were transferred to a 96-well V-bottom plate at 1–2 million cells/well and stimulated for 6 h with 2 µg/mL consensus subtype B envelope peptide pool (NIH AIDS Repository) in the presence of protein transport inhibitor (BD Golgi Plug™ containing Brefeldin A, 1 μg/mL, and BD Golgi Stop™ containing monensin, 1 μg/mL, BD Biosciences). For the positive control, cells were stimulated with eBiosciences stimulation cocktail containing PMA and Ionomycin (eBioscience™ Cell Stimulation Cocktail; 00-4970-03) while the negative controls received an equal concentration of DMSO as present in the peptides. After the incubation period, cells were washed and stained with LIVE/DEAD Fixable Aqua Dead Cell Stain Kit (Invitrogen/Thermo Fisher Scientific, 1:1000), followed by surface staining with a cocktail of fluorescently labeled antibodies specific for Tfh cells for 30 min at 4 °C, washed twice with FACS buffer and then fixed/permeabilized for 40 min at 4 °C in the dark using the eBioscience™ Intracellular Fixation and Permeabilization Buffer Set (Thermo Fisher Scientific, 88-8824-00) as per the manufacturer’s instructions. Cells were then incubated with an intracellular antibody cocktail (Table S9) for 30 min at 4 °C, washed twice with permeabilization buffer, and resuspended in FACS buffer. Appropriate single-color compensation controls and Fluorescence minus one control (FMO) were prepared simultaneously and were included in each analysis. Flow cytometry analysis was performed on a BD LSR II flow cytometer, and data were acquired using Diva software (BD Biosciences). The results were analyzed using FlowJo software version 10.7.1 (BD Biosciences). In each analysis, respective FMO controls were used to set up the gates or to identify the positive populations. The gating strategy applied for the evaluation of flow cytometry-acquired data were provided in Fig. S4B, C. The flow cell panel for intracellular cytokines and Tfh cells are shown in Table S9.

### Statistical analysis

Data were presented as arithmetic mean ± SEM or geometric mean ± geometric SD. Group comparisons were conducted using the Kruskal–Wallis test with Dunn’s post hoc analysis for multiple comparisons or a two-tailed Mann–Whitney test as detailed in the figure legends. A *p* value of <0.05 was considered statistically significant. Tests for statistical difference between group decay rates were calculated by ANOVA. Pairwise differences between titers for each feature (respectively) were calculated with Fisher’s least square difference method. The AUCs between two groups in the GTL and LUC ADCC assays were compared using the Wilcoxon test. Statistical analysis of differential gene expression in RNAseq data were performed using the DESeq2 package. The significance threshold for the identification of differentially expressed genes, corrected for multiple testing, was set as an adjusted *p* value <0.05. GSEA was carried out with the GSEA software, calculating a normalized enrichment score and FDR. An FDR<0.05 was considered significant. For the heat maps, the average assay-specific Z-scores for each treatment group within each timepoint was calculated. Heat maps were constructed for the normalized measures of each of the two datasets (Humoral and Cellular Response) across the respective available timepoints. Heat maps were constructed using R statistical software v4.4.1 (R package *ggplot2*). Data presented in this study represent biological replicates. Analyses were performed using GraphPad PRISM version 10.2.2.

## Supplementary information

Combined Supplementary figures and tables 30 Oct 2025

## Data Availability

All data associated with this study are present in the paper or in the Supplementary Materials. RNAseq generated in this study were submitted to the National Center for Biotechnology Information Gene Expression Omnibus (GEO) repository under accession number GSE308512.
