# Supplementary material for: ALFQ adjuvanted HIV-1 envelope protein vaccination elicits durable functional antibody and cellular responses in nonhuman primates
Source: NPJ Vaccines. 2025 Dec 19;11:1. doi: 10.1038/s41541-025-01322-7 (PMC12764542; doi:10.1038/s41541-025-01322-7)
Supplement: Supplementary file 1 — Combined Supplementary figures and tables 30 Oct 2025 [file 41541_2025_1322_MOESM1_ESM.pdf]

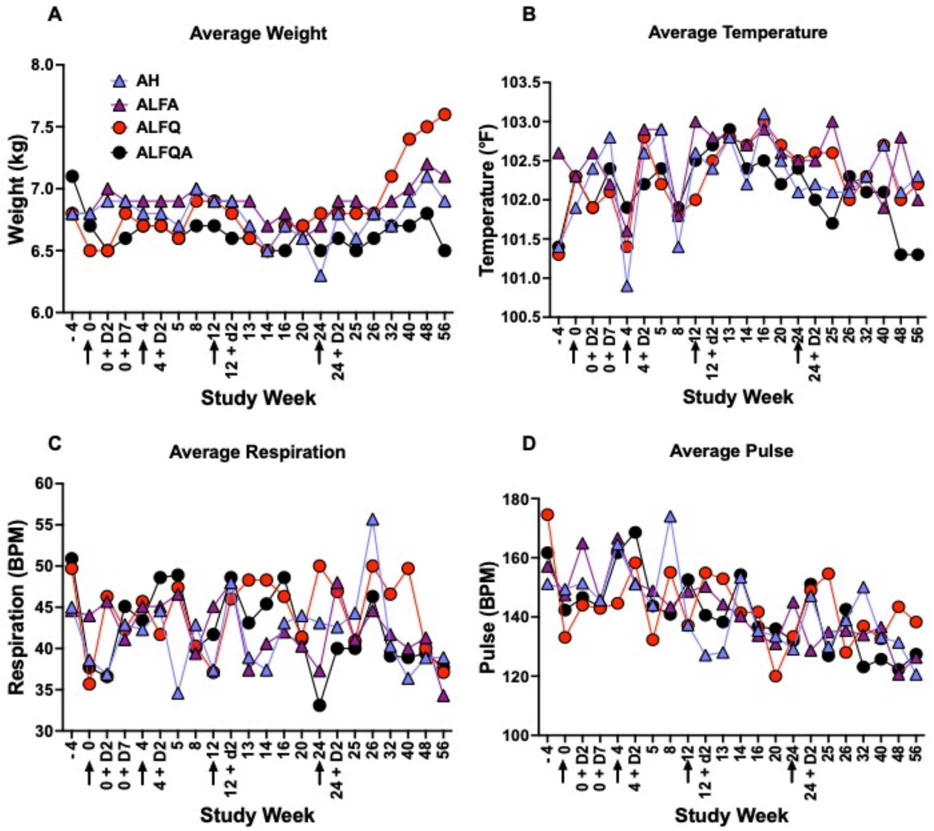

Supplementary Figure 1. Physical parameters following vaccination. The average (n = 7/group) (**A**) weight (**B**) temperature (**C**) respiration and (**D**) pulse from each of the adjuvant groups at all the time points during the study period are shown.

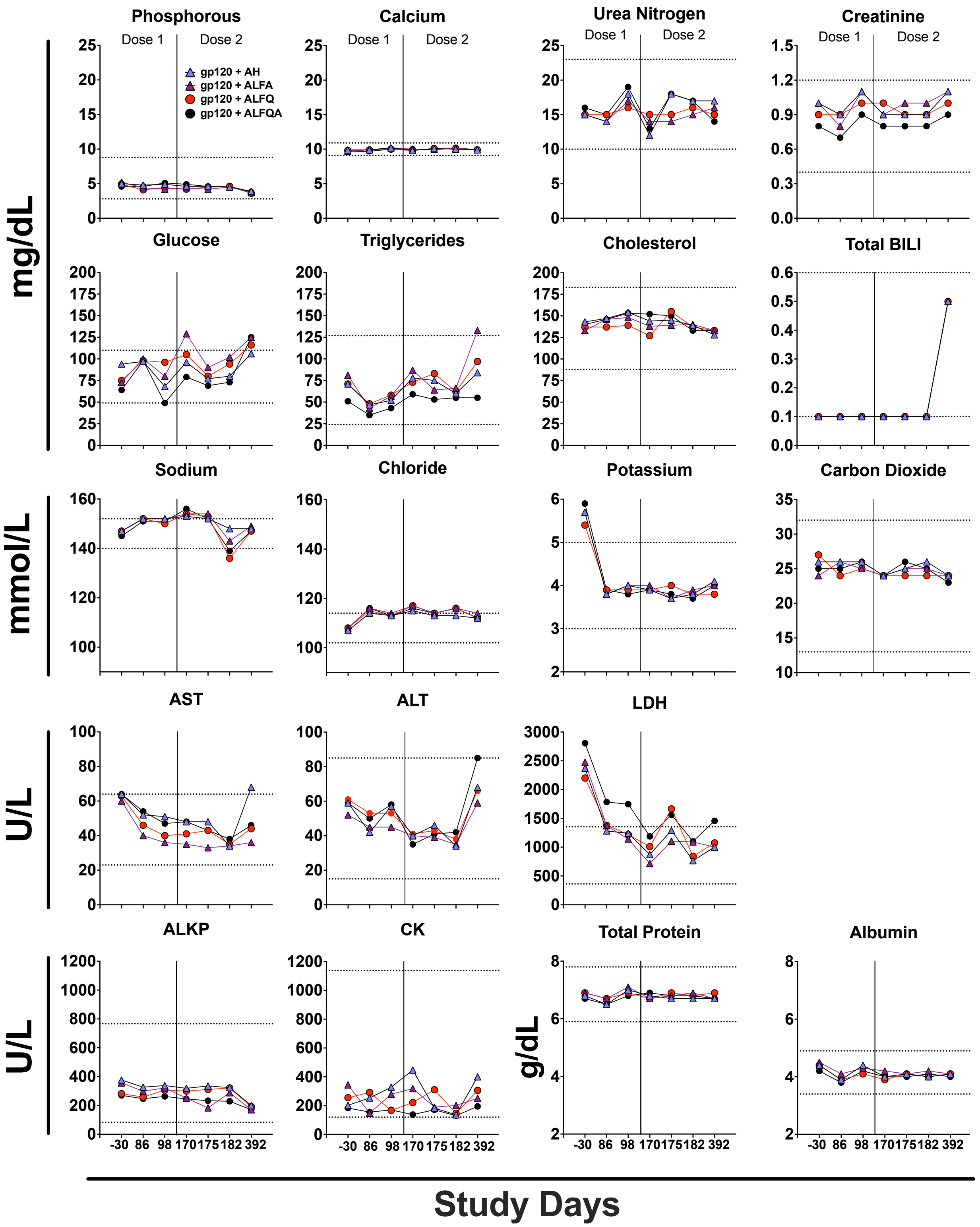

Supplementary Figure 2. Mean Blood Chemistry following vaccination. The average (n = 7/group) parameters for the various blood chemistry components during the study days for the 4 vaccination groups are shown. The dotted lines represent the upper and lower limits. Doses 1 and dose 2 represent vaccine doses ALVAC + gp120 + adjuvant. Samples were analyzed 2 days (day

86), 2 weeks (day 98) after the first vaccine dose; 2 days (day 170), 1 week (day 175), 2 weeks (day 182), and 30 weeks (day 392) after the second dose. The vertical solid line separates the two vaccine doses administered.

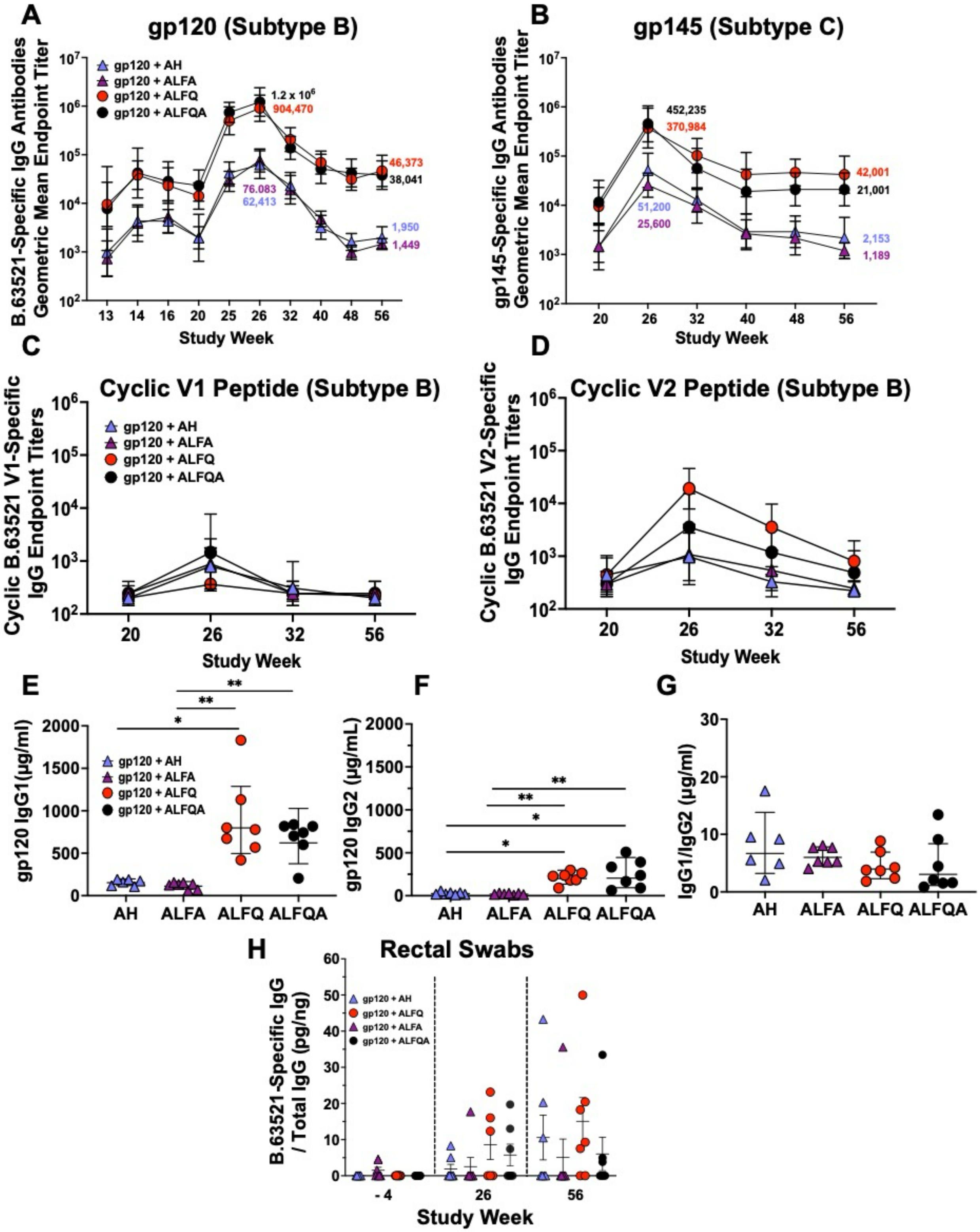

Supplementary Figure 3. Time course of the antibody responses following vaccination. The longitudinal geometric mean antibody end point titers specific for (**A**) B.63521 gp120, (**B**) gp145 acute C and (**C**) subtype B cyclic V1 peptide and (**D**) cyclic V2 peptide (n= 7/group, assayed in

triplicate) by ELISA following vaccination are shown. B.63521 gp120 protein-specific (**E**) IgG1 and (**F**) IgG2 subclasses for each group was quantified as µg/mL (**G**) The ratio of IgG1 to IgG2. The data are represented as geometric mean endpoint titers with geometric SD. (**H)** B.63521 gp120 protein-specific IgG and total IgG in the rectal swab extracts were measured for each animal (n=7/group) by surface plasmon resonance. The graph depicts the ratio of B.63521-specific IgG to the total IgG in the rectal swab extracts in pg/ng as measured by surface plasmon resonance. Each dot in all the panels represents the average for triplicate measurements from each animal at the time points indicated. Statistical comparisons between different adjuvant groups were performed using Kruskal-Wallis test with Dunn's multiple comparison test. *P < 0.04, **P < 0.009, and ***P < 0.0006.

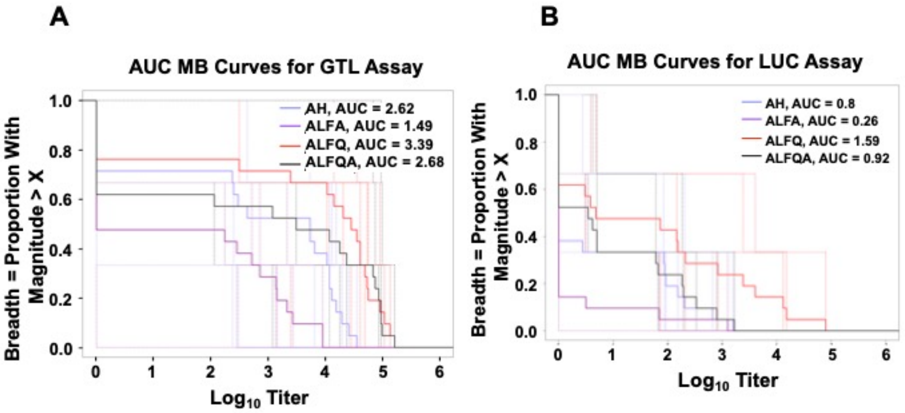

Supplementary Figure 4. Plasma samples from individual NHPs for each individual adjuvant formulation was assessed at study week 26 for antibody-dependent cytotoxic cell activity (ADCC) with gp120-coated target cells, (**A**) A magnitude-breadth curve was used to describe the ADCC titer response and breadth (number of isolates targeted) of an individual sample assayed against three different gp120s for the GTL assay or (**B**) a panel of three different HIV-1 IMCs for the Luc assay. The x-axis represents the threshold of ADCC response that is considered positive for the magnitude-breadth for the three proteins, or three IMCs tested, whereas the y-axis represents the percent of the three targets showing ADCC responses. The area under the curve (AUC) of a magnitude-breadth curve equals the average ADCC response over the three targets tested for each of the assays. The AUCs between two groups were compared using Wilcoxon test. In the GTL assay, the AUC for titer in the group vaccinated with vaccine-ALFQ significantly differed from AUC for titer in the group vaccinated with vaccine-ALFA (p-value = 0.0152). None of the other groups showed any significance (p > 0.2013). In the Luc assay, the AUC for titer in the group vaccinated with vaccine-ALFQ significantly differed from AUC for titer in the group vaccinated with vaccine-ALFA (p-value = 0.0130), None of the other groups showed any significance (p > 0.1248).

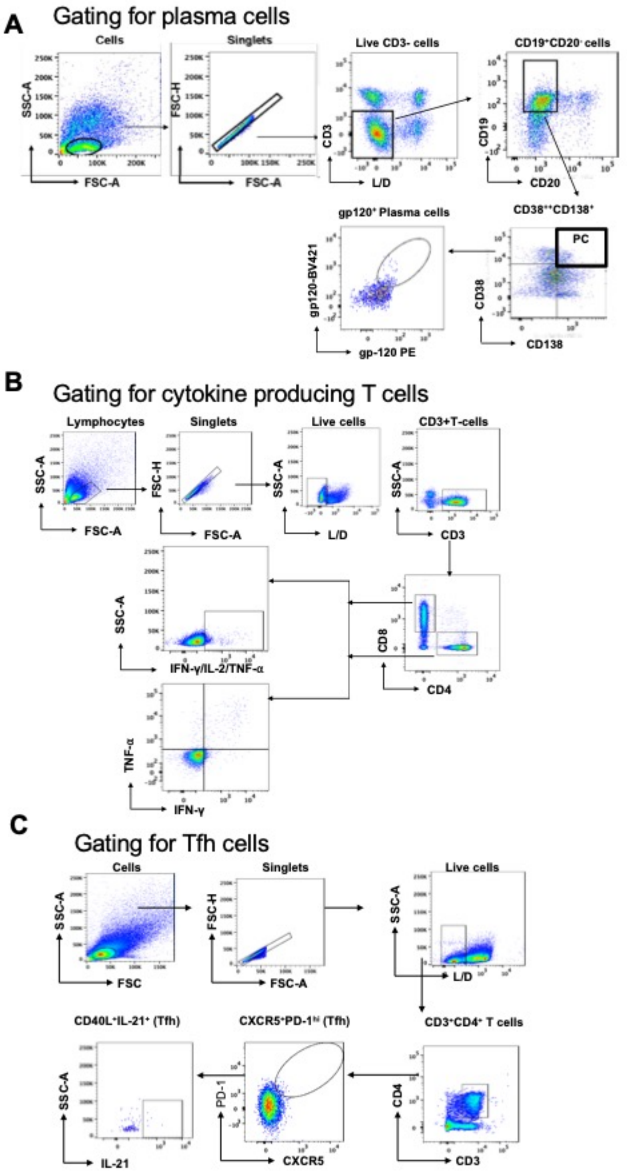

Supplementary Figure 5. Flow gating strategies in PBMCs. Gating strategy for (**A**) bone marrow plasma cells (**B**) cytokine producing cells, and (**C**) T follicular helper cells (Tfh).

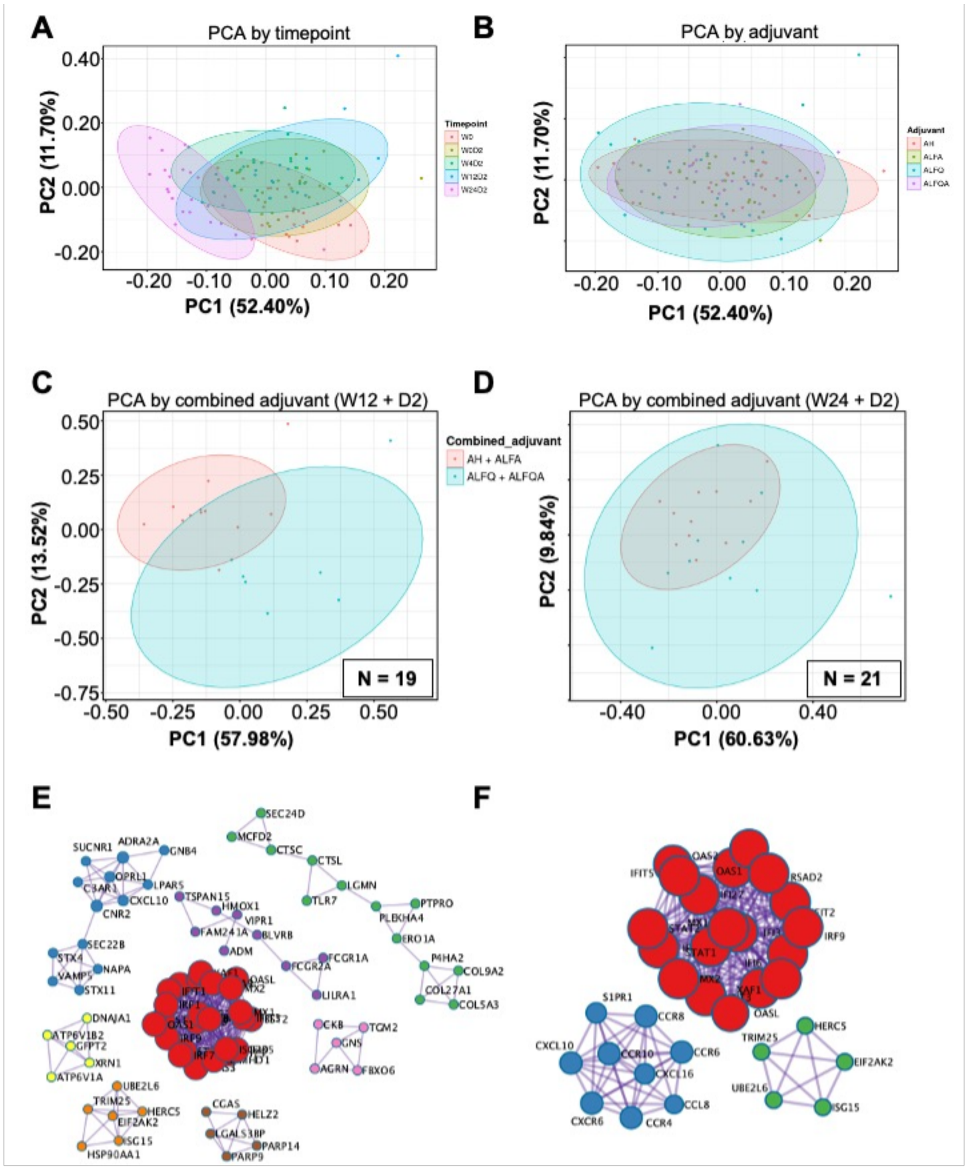

Supplementary Figure 6. Principal component and protein-protein interaction enrichment analysis. Principal component analysis (PCA) of all samples colored by (**A**) timepoint and (**B**) adjuvant. Ellipses indicate groups by adjuvant and timepoint respectively. Principal component analysis (PCA) of all samples colored by combined adjuvants (**C**) AH/ALFA and (**D**) ALFQ/ALFQA vaccine formulations at Week 12 + Day 2 and Week 24 + Day 2 timepoints. The center represents the centroid of the PCA coordinates for points (samples) within them; and the boundaries indicate maximum variance from the centroid at 95% confidence interval. Protein-protein interaction enrichment analysis using DEG (FDR <0.05, absolute (Fold change) > 1.5) as implemented in Metascape identifies highly interconnected MCODE clusters for (**E**) study week 12 + day 2 and (**F**) study week 24 + day 2 comparisons between ALFQ/ALFQA versus AH/ALFA arms. Colors indicate different clusters of nodes and sizes indicate the number of connections with other nodes in the network. (FDR = False discovery rate). See Tables S11 and S12 for cluster annotation.

Table S1. Blood Cell count parameters in NHPs vaccinated with Vaccine-AH

			**Vaccine-AH Adjuvant Dose 1**		**Vaccine-AH Adjuvant Dose 2**		
		Pre Immune Baseline	Day 0 (Study day 84)	Day 7 (Study day 91)	Day 0 (Study day 168)	Day 7 (Study day 175)	Day 392 (Study End)
**CBC**	Reference Range	Average ± S.D.	Average ± S.D.	Average ± S.D.	Average ± S.D.	Average ± S.D.	Average ± S.D.
**WBC**	4.00-13.00	9.59 ± 4.66	7.67 ± 1.90	7.94 ± 0.81	8.26 ± 3.35	8.90 ± 3.65	6.64 ± 3.01
**RBC**	4.30-6.20	5.54 ± 0.22	5.60 ± 0.38	5.37 ± 0.25	5.44 ± 0.38	5.19 ± 0.28	5.25 ± 0.32
**HGB**	10.6-14.2	12.9 ± 0.6	12.9 ± 0.8	12.4 ± 0.6	12.6 ± 0.9	12.0 ± 0.8	12.4 ± 0.9
**HCT**	32.6-43.8	39.5 ± 1.7	40.8 ± 2.4	39.3 ± 1.8	38.5 ± 2.9	37.0 ± 2.5	37.9 ± 3.1
**MCV**	65.6-76.9	71.3 ± 2.1	72.9 ± 2.5	73.2 ± 2.4	70.9 ± 2.4	71.3 ± 2.3	72.1 ± 2.3
**MCH**	20.6-24.6	23.4 ± 0.5	23.1 ± 0.5	23.1 ± 0.4	23.1 ± 0.4	23.1 ± 0.5	23.6 ± 0.5
**MCHC**	30.6-32.8	32.8 ± 0.5	31.7 ± 0.7	31.6 ± 0.7	32.7 ± 0.8	32.4 ± 0.6	32.7 ± 0.7
**PLT**	97-436	270 ± 111	397 ± 87	404 ± 27	249 ± 86	392 ± 44	264 ± 42
**RDW-CV**	12.3-15	13 ± 0.5	13 ± 0.7	13 ± 0.7	13 ± 0.3	13 ± 0.4	13 ± 0.4
**MPV**	9.2-12.4	11.8 ± 1.1	11.4 ± 0.9	11.0 ± 0.6	11.8 ± 0.9	11.0 ± 0.6	11.8 ± 0.8
**NEUT%**	22.5-81.6	70.6 ± 12.5	66.8 ± 5.7	66.3 ± 5.4	75.5 ± 6.5	78.0 ± 10.7	72.6 ± 9.3
**LYMPH%**	7.4-52.1	24.7 ± 11.5	29.6 ± 4.6	28.5 ± 6.1	19.6 ± 6.9	24.8 ± 15.8	25.9 ± 7.1
**MONO%**	1.7-7.2	4.3 ± 1.8	4.7 ± 1.1	4.3 ± 0.6	4.3 ± 0.9	3.7 ± 1	4.1 ± 1.4
**EO%**	0.0-2.6	0.3 ± 0.5	0.2 ± 0.5	0.2 ± 0.2	0.1 ± 0.2	0.1 ± 0.2	0.1 ± 0.2
**BASO%**	0.0-1.0	0.2 ± 0.1	0.2 ± 0.1	0.2 ± 0.1	0.1 ± 0.1	0.1 ± 01	0.1 ± 0.1
**NEUT#**	1.25-9.20	7.28 ± 4.41	5.14 ± 1.40	4.92 ± 1.02	6.43 ± 3.35	6.59 ± 3.80	4.24 ± 2.12
**LYMPH#**	0.80-3.71	2.12 ± 0.80	2.14 ± 0.67	2.13 ± 0.69	1.66 ± 0.51	1.88 ± 0.76	1.34 ± 0.90
**MONO#**	0.13-0.63	0.39 ± 0.16	0.36 ± 0.12	0.34 ± 0.10	0.37 ± 0.18	0.38 ± 0.21	0.21 ± 0.08
**EO#**	0.00-0.18	0.02 ± 0.02	0.02 ± 0.03	0.03 ± 0.05	0.01 ± 0.01	0.01 ± 0.02	0.01 ± 0.01
**BASO#**	0.00-0.01	0.01 ± 0.01	0.01 ± 0.01	0.02 ± 0.01	0.01 ± 0.01	0.01 ± 0.00	0.004 ± 0.01
**RET%**	0.40-1.40	0.65 ± 0.11	0.64 ± 0.16	0.79 ± 0.14	0.84 ± 0.15	0.90 ± 0.25	0.62 ± 0.10
**RET#**	0.02-0.08	0.04 ± 0.01	0.04 ± 0.01	0.04 ± 0.01	0.04 ± 0.01	0.05 ± 0.01	0.03 ± 0.01

Table S2. Blood Cell count parameters in NHPs vaccinated with Vaccine-ALFA

			**Vaccine-AH Adjuvant Dose 1**		**Vaccine-AH Adjuvant Dose 2**		
		Pre Immune Baseline	Day 0 (Study day 84)	Day 7 (Study day 91)	Day 0 (Study day 168)	Day 7 (Study day 175)	Day 392 (Study End)
**CBC**	Reference Range	Average ± S.D.	Average ± S.D.	Average ± S.D.	Average ± S.D.	Average ± S.D.	Average ± S.D.
**WBC**	4.00-13.00	8.18 ± 4.4	6.46 ± 2.14	6.81 ± 2.67	5.56 ± 2.10	7.43 ± 3.08	6.33 ± 2.46
**RBC**	4.30-6.20	5.49 + 0.43	5.34 ± 0.49	5.08 ± 0.46	5.39 ± 0.38	5.12 ± 0.38	5.23 ± 0.53
**HGB**	10.6-14.2	12.69 ± 1.2	12.1 ± 1.4	11.5 ± 1.39	12.2 ± 1.3	11.5 ± 1.40	12.0 ± 1.56
**HCT**	32.6-43.8	39.58 ± 3.5	39.2 ± 4	37.4 ± 4.3	38.1 ± 3.9	36.5 ± 4.2	37.9 ± 4.5
**MCV**	65.6-76.9	72.13 ± 2.2	74.7 ± 3.2	73.6 ± 5.0	70.7 ± 4.7	71.1 ± 4.7	72.6 ± 5.3
**MCH**	20.6-24.6	23.12 ± 0.7	22.6 ± 1.3	22.7 ± 1.5	22.6 ± 1.6	22.5 ± 1.7	23.0 ± 1.9
**MCHC**	30.6-32.8	32.05 ± 0.5	30.8 ± 0.6	30.8 ± 0.5	32.0 ± 0.7	31.6 ± 1.2	31.7 ± 0.9
**PLT**	97-436	296.69 ± 83	343 ± 92	386 ± 37	284 ± 59	339 ± 90	230 ± 71
**RDW-CV**	12.3-15	13.02 ± 0.6	13.3 ± 0.7	13.2 ± 0.9	14.1 ± 1.6	14.2 ± 1.8	13.1 ± 1.3
**MPV**	9.2-12.4	11.85 ± 0.7	11.5 ± 0.9	11.3 ± 0.6	11.9 ± 0.8	11.7 ± 1.0	11.6 ± 0.7
**NEUT%**	22.5-81.6	60.74 ± 11	60.8 ± 10.3	55.2 ± 6.4	57.2 ± 9.3	66.3 ± 14.5	67.1 ± 9.8
**LYMPH%**	7.4-52.1	33.85 ± 9.6	32.8 ± 9.6	37.5 ± 6.1	36.5 ± 9	28.7 ± 13.3	27.6 ± 9.3
**MONO%**	1.7-7.2	4.99 ± 1.7	5.5 ± 1.7	5.7 ± 1.2	5.9 ± 0.9	4.8 ± 1.67	4.8 ± 0.6
**EO%**	0.0-2.6	0.26 ± 0.5	0.8 ± 1.0	0.9 ± 1.3	0.3 ± 0.3	0.1 ± 0.2	0.4 ± 0.5
**BASO%**	0.0-1.0	0.16 ± 0.1	0.2 ± 0.2	0.2 ± 0.1	0.1 ± 0.1	0.1 ± 0.1	0.1 ± 0.1
**NEUT#**	1.25-9.20	5.35 ± 3.65	4.45 ± 1.82	3.68 ± 1.22	3.72 ± 1.89	5.00 ± 2.85	4.34 ± 2.03
**LYMPH#**	0.80-3.71	2.66 ± 1.28	2.40 ± 1.24	2.49 ± 1.26	2.22 ± 0.91	2.22 ± 1.10	1.66 ± 0.67
**MONO#**	0.13-0.63	0.38 ± 0.19	0.39 ± 0.18	0.43 ± 0.13	0.38 ± 0.19	0.39 ± 0.18	0.30 ± 0.13
**EO#**	0.00-0.18	0.02 ± 0.03	0.06 ± 0.07	0.08 ± 0.13	0.02 ± 0.03	0.01 ± 0.02	0.02 ± 0.03
**BASO#**	0.00-0.01	0.01 ± 0.01	0.013 ± 0.01	0.014 ± 0.01	0.01 ± 0.01	0.01 ± 0.01	0.004 ± 0.01
**RET%**	0.40-1.40	0.72 ± 0.14	0.86 ± 0.32	1.03 ± 0.15	0.98 ± 0.12	1.17 ± 0.24	0.70 ± 0.11
**RET#**	0.02-0.08	0.04 ± 0.01	0.051 ± 0.01	0.05 ± 0.09	0.05 ± 0.01	0.06 ± 0.01	0.04 ± 0.01

Table S3. Blood Cell count parameters in NHPs vaccinated with Vaccine-ALFQ

			**Vaccine-AH Adjuvant Dose 1**		**Vaccine-AH Adjuvant Dose 2**		
		Pre Immune Baseline	Day 0 (Study day 84)	Day 7 (Study day 91)	Day 0 (Study day 168)	Day 7 (Study day 175)	Day 392 (Study End)
**CBC**	Reference Range	Average ± S.D.	Average ± S.D.	Average ± S.D.	Average ± S.D.	Average ± S.D.	Average ± S.D.
**WBC**	4.00-13.00	7.20 ± 2.08	6.60 ± 0.98	6.76 ± 1.47	5.76 ± 1.59	8.17 ± 2.04	7.88 ± 1.53
**RBC**	4.30-6.20	5.39 ± 0.57	5.40 ± 0.40	5.11 ± 0.35	5.44 ± 0.36	5.21 ± 0.26	5.26 ± 0.41
**HGB**	10.6-14.2	12.8 ± 1.1	12.5 ± 0.7	11.8 ± 0.6	12.7 ± 0.5	12.1 ± 0.4	12.6 ± 0.8
**HCT**	32.6-43.8	39.8 ± 3.2	40.7 ± 2.0	38.7 ± 1.5	40.0 ± 1.4	38.6 ± 1.3	39.4 ± 1.9
**MCV**	65.6-76.9	74.0 ± 3.2	75.5 ± 3.2	76.0 ± 3.1	73.7 ± 3.4	74.3 ± 3	75.1 ± 3.3
**MCH**	20.6-24.6	23.7 ± 0.8	23.2 ± 0.7	23.1 ± 0.6	23.3 ± 0.9	23.2 ± 1.1	24.0 ± 0.8
**MCHC**	30.6-32.8	32.1 ± 0.6	30.7 ± 0.5	30.4 ± 0.7	31.6 ± 0.4	31.2 ± 0.5	32.0 ± 0.7
**PLT**	97-436	255 ± 109	352 ± 41	455 ± 88	276 ± 67	386 ± 114	264 ± 85
**RDW-CV**	12.3-15	13 ± 0.0	13 ± 0.0	13 ± 1	13 ± 0.0	13 ± 1	13 ± 0.0
**MPV**	9.2-12.4	12.2 ± 1.2	11.5 ± 1.1	11.2 ± 1	12.1 ± 0.9	11.8 ± 1	11.7 ± 1.1
**NEUT%**	22.5-81.6	66.7 ± 10.6	63.3 ± 7.0	55.6 ± 10.5	59.4 ± 9.4	57.6 ± 9.7	76.4 ± 7.6
**LYMPH%**	7.4-52.1	28.5 ± 9.6	31.3 ± 5.6	41.0 ± 7.4	34.7 ± 9	37.4 ± 8.4	19.8 ± 6.4
**MONO%**	1.7-7.2	4.2 ± 1.6	4.8 ± 1.4	5.2 ± 1.1	4.8 ± 1.2	3.9 ± 1	3.4 ± 1.2
**EO%**	0.0-2.6	0.4 ± 0.3	0.3 ± 0.3	0.6 ± 0.6	0.1 ± 0.1	0.1 ± 0.1	0.3 ± 0.3
**BASO%**	0.0-1.0	0.2 ± 0.2	0.3 ± 0.3	0.4 ± 0.3	0.2 ± 0.2	0.1 ± 0.1	0.0 ± 0.1
**NEUT#**	1.25-9.20	5.10 ± 2.03	4.17 ± 0.64	4.30 ± 1.8	3.36 ± 0.77	4.18 ± 0.70	5.67 ± 1.56
**LYMPH#**	0.80-3.71	1.99 ± 0.56	2.07 ± 0.54	2.80 ± 0.9	1.66 ± 0.53	2.13 ± 0.73	1.48 ± 0.60
**MONO#**	0.13-0.63	0.31 ± 0.15	0.32 ± 0.13	0.40 ± 0.20	0.35 ± 0.18	0.29 ± 0.06	0.25 ± 0.10
**EO#**	0.00-0.18	0.03 ± 0.02	0.02 ± 0.02	0.04 ± 0.03	0.01 ± 0.01	0.00 ± 0.01	0.02 ± 0.03
**BASO#**	0.00-0.01	0.01 ± 0.01	0.02 ± 0.01	0.03 ± 0.02	0.01 ± 0.01	0.01 ± 0.00	0.003 ± 0.00
**RET%**	0.40-1.40	0.68 ± 0.20	0.76 ± 0.33	0.98 ± 0.23	0.80 ± 0.23	1.16 ± 0.26	0.80 ± 0.36
**RET#**	0.02-0.08	0.04 ± 0.01	0.04 ± 0.005	0.05 ± 0.012	0.04 ± 0.012	0.06 ± 0.01	0.04 ± 0.005

Table S4. Blood Cell count parameters in NHPs vaccinated with Vaccine-ALFQA

			**Vaccine-AH Adjuvant Dose 1**		**Vaccine-AH Adjuvant Dose 2**		
		Pre Immune Baseline	Day 0 (Study day 84)	Day 7 (Study day 91)	Day 0 (Study day 168)	Day 7 (Study day 175)	Day 392 (Study End)
**CBC**	Reference Range	Average ± S.D.	Average ± S.D.	Average ± S.D.	Average ± S.D.	Average ± S.D.	Average ± S.D.
**WBC**	4.00-13.00	6.96 ± 2.27	6.37 ± 1.40	7.53 ± 1.10	8.84 ± 3.58	10.32 ± 4.75	5.36 ± 1.51
**RBC**	4.30-6.20	5.13 ± 0.54	5.36 ± 0.43	5.15 ± 0.34	5.28 ± 0.12	5.07 ± 0.21	5.06 ± 0.39
**HGB**	10.6-14.2	11.9 ± 1.2	12.3 ± 1.0	11.8 ± 0.9	12.0 ± 0.5	11.6 ± 0.6	11.9 ± 0.9
**HCT**	32.6-43.8	37.1 ± 3.7	39.7 ± 2.2	38.0 ± 1.7	38.2 ± 1.0	36.7 ± 1.1	37.1 ± 1.9
**MCV**	65.6-76.9	72.4 ± 2.6	74.2 ± 2.6	74.0 ± 3.1	72.4 ± 2.3	72.4 ± 2.5	73.5 ± 3.5
**MCH**	20.6-24.6	23.1 ± 0.3	23.0 ± 0.2	22.9 ± 0.3	22.8 ± 0.8	22.8 ± 0.4	23.4 ± 0.4
**MCHC**	30.6-32.8	32.0 ± 1.2	31.0 ± 1.0	31.0 ± 1.2	31.5 ± 0.8	31.5 ± 1.0	31.9 ± 1.4
**PLT**	97-436	260 ± 107	330 ± 44	393 ± 73	312 ± 93	383 ± 88	223 ± 66
**RDW-CV**	12.3-15	12.8 ± 0.7	12.7 ± 0.5	12.7 ± 0.5	13.0 ± 0.9	12.7 ± 0.4	12.4 ± 0.5
**MPV**	9.2-12.4	11.9 ± 1.0	11.7 ± 0.7	11.7 ± 0.4	12.0 ± 1.0	11.6 ± 0.7	11.9 ± 0.9
**NEUT%**	22.5-81.6	56.6 ± 13.9	59.2 ± 15.8	60.3 ± 14.6	65.5 ± 14.3	67.4 ± 17.7	61.7 ± 10.4
**LYMPH%**	7.4-52.1	37.9 ± 12.7	35.3 ± 14.2	34.3 ± 13.2	37.9 ± 11.1	35.3 ± 8.8	26.4 ± 13.6
**MONO%**	1.7-7.2	4.4 ± 1.2	4.6 ± 1.3	4.1 ± 1.4	3.6 ± 1.6	4.2 ± 1.7	3.6 ± 1.8
**EO%**	0.0-2.6	0.9 ± 1.1	0.4 ± 0.6	1.0 ± 1.5	0.9 ± 1.4	0.1 ± 0.2	0.8 ± 1.0
**BASO%**	0.0-1.0	0.1 ± 0.1	0.1 ± 0.1	0.3 ± 0.1	0.1 ± 0.0	0.1 ± 0.1	0.0 ± 0.1
**NEUT#**	1.25-9.20	4.16 ± 2.14	3.54 ± 1.29	4.59 ± 1.56	4.11 ± 1.16	7.67 ± 5.36	4.38 ± 2.5
**LYMPH#**	0.80-3.71	2.43 ± 0.63	2.33 ± 0.91	2.55 ± 1.04	2.37 ± 0.80	2.22 ± 0.67	1.37 ± 0.49
**MONO#**	0.13-0.63	0.30 ± 0.08	0.30 ± 0.05	0.30 ± 0.08	0.31 ± 0.11	0.37 ± 0.11	0.19 ± 0.07
**EO#**	0.00-0.18	0.06 ± 0.06	0.05 ± 0.07	0.07 ± 0.1	0.06 ± 0.09	0.05 ± 0.11	0.04 ± 0.05
**BASO#**	0.00-0.01	0.01 ± 0.00	0.01 ± 0.01	0.021 ± 0.01	0.010 ± 0.00	0.009 ± 0	0.001 ± 0.00
**RET%**	0.40-1.40	0.66 ± 0.20	0.69 ± 0.27	0.94 ± 0.52	0.66 ± 0.17	0.92 ± 0.31	0.63 ± 0.23
**RET#**	0.02-0.08	0.03 ± 0.01	0.04 ± 0.02	0.05 ± 0.03	0.04 ± 0.01	0.05 ± 0.02	0.03 ± 0.01

Table S5. Pairwise comparison of decay rates for each of the binding antibody titers

Pairwise Group	Mean Difference	95% CI		P Value	Mean Difference	95% CI		P value
AH-ALFA	0.4821	-0.1519	1.1161	ns	0.3466	-1.0043	1.6974	ns
AH-ALFQ	-0.254	-0.888	0.38	ns	-0.1484	-1.4993	1.2024	ns
AH-ALFQA	-0.363	-0.997	0.271	ns	-0.6434	-1.9943	0.7074	ns
ALFQ-ALFA	0.7361	0.1021	1.3701	p<0.05	0.495	-0.8559	1.8459	ns
ALFQ-ALFQA	-0.109	-0.743	0.525	ns	-0.495	-1.8459	0.8559	ns
ALFQA-ALFA	0.8451	0.2111	1.4791	p<0.05	0.99	-0.3609	2.3409	ns

Table S6. Summary of GTL responses to gp120-coated targets at study week 26

		B.63521				MN				TV1			
		N	Mean	Median	Std Dv	N	Mean	Median	Std Dv	N	Mean	Median	Std Dv
**Group 1**	Peak GTL	6	8.94	8.61	1.26	5	14.04	12.71	4.69	4	7.37	6.61	2.13
	GTL Titer	6	14569.33	12636.16	14098.99	5	13116.74	12013.15	5259.71	4	2937.76	2945.58	2971.11
	%NKs cells	6	25.80	27.05	4.12	5	19.40	18.00	5.46	4	13.93	12.85	2.75
**Group 2**	Peak GTL	2	6.20	6.20	0.92	6	9.72	8.34	4.06	2	7.14	7.14	0.59
	GTL Titer	2	444.53	444.53	381.59	6	4024.42	2080.23	4036.89	2	1345.58	1345.58	1159.36
	%NKs cells	2	24.60	24.60	12.59	6	15.10	12.90	4.68	2	13.20	13.20	0.85
**Group 3**	Peak GTL	4	12.09	12.04	3.15	7	12.82	12.14	4.82	5	11.80	11.10	5.14
	GTL Titer	4	42888.42	30931.49	49376.03	7	72488.41	41769.63	60336.70	5	37170.25	36372.40	19681.39
	%NKs cells	4	26.28	17.20	18.22	7	18.33	16.70	4.89	5	18.36	18.50	5.34
**Group 4**	Peak GTL	3	7.09	7.89	1.56	6	8.64	8.18	3.07	4	9.71	9.21	4.98
	GTL Titer	3	57485.96	75157.15	50892.80	6	62322.22	46580.37	58486.17	4	49701.15	49908.87	54854.62
	%NKs cells	3	40.03	29.10	23.86	6	14.08	13.45	2.99	4	16.55	16.25	5.17

Results are reported for responses greater than or equal to 5% Granzyme B activity in target cells after background subtraction.

Table S7. Summary responses at study week 26 for luciferase activity normalized to baseline

		96ZM				WITO				TV1			
		**N**	**Mean**	**Median**	**Std Dv**	**N**	**Mean**	**Median**	**Std Dv**	**N**	**Mean**	**Median**	**Std Dv**
**Group 1**	Peak GTL	1	13.37	13.17	n/a	4	19.45	17.96	7.42	3	12.56	11.37	2.43
	GTL Titer	1	51.36	51.36	n/a	4	243.10	120.07	271.14	3	621.53	199.62	835.52
**Group 2**	Peak GTL	1	14.91	14.91	n/a	0				2	11.10	11.10	1.53
	GTL Titer	1	146.40	146.40	n/a	0				2	662.29	662.29	838.39
**Group 3**	Peak GTL	3	18.87	18.92	1.88	7	21.49	22.7	4.60	3	18.41	18.50	5.12
	GTL Titer	3	1941.84	651.90	2729.13	7	14033.93	841.62	29364.30	3	5957.26	2442.99	8161.66
**Group 4**	Peak GTL	4	12.40	11.68	2.52	5	20.78	19.03	4.04	2	12.81	12.81	1.20
	GTL Titer	4	4891.26	4819.58	4767.01	5	606.50	336.59	645.49	2	121.03	121.03	79.41

Table S8. Plasma cell staining panel (Bone Marrow)

Fluorochrome	Antigen	Clone	Manufacturer	Cat#
**Surface**				
APC-Cy7	Fixable Far Red Dead Cell Stain Kit	All species	ThermoFischer Scientific	L10120
PE-Cy7	CD19	J3-119	Beckman Coulter	IM3628U
Brilliant Violet 510	CD20	2H7	BioLegend	302340
APC	CD38	OK10	NHP Reagents resources	AB_2819277
FITC	CD138 (Syndecan 1)	DL-101	BioLegend	352304
Alexa Fluor 700	CD3	SP34-2	BD Biosciences	557917
BV421	B.65321 gp120		In-house labeling	-
PE	B.65321 gp120		In-house labeling	-

Table S9. T cell intracellular cytokine and T follicular helper cell staining panel (PBMCs)

Fluorochrome	Antigen	Clone	Manufacturer	Cat#
**Surface**				
Aqua/BV510	Live/Dead Fixable Aqua Dead Cell Stain Kit	All species	L34966	L34966
APC-Cy7	CD3	SP34-2	BD Biosciences	557757
Brilliant Violet 605	CD4	L200	BD Biosciences	562843
BUV395	CD8	RPA-T8	BD Biosciences	563795
Brilliant Violet 650	CCR5	3A9	BD Biosciences	564999
APC	CD185 (CXCR5)	MU5UBEE	Thermo Fisher Scientific	17-9185-41
Brilliant Violet 785	PD-1	EH12.2H7	BioLegend	329930
**Intracellular/Intranuclear**				
Brilliant Violet 421	IFN-gamma	B27	BioLegend	506538
Alexa Fluor 488	TNF alpha	MAb11	BioLegend	502915
Brilliant Violet 750	IL-2	MQ1-17H12	BD Biosciences	566361
PE	IL-21	3A3-N2	BioLegend	513004

Table S10. Comparison of DEGs for the four vaccine-adjuvant arms two days after the first and second protein boosts

Comparison	Timepoint	Significant DEGs
AH v/s ALFA	Week 12 + day 2	0
AH v/s ALFA	Week 24 + day 2	1 (SLC15A1)
ALFQ v/s ALFQA	Week 12 + day 2	3 (ADM, CTSL, USHBP1)
ALFQ v/s ALFQA	Week 24 + day 2	2 (CCNJ, TBC1D2)
Total		6

Table S11. Enriched Metascape pathways using significant DEGs identified at week 12

Color	MCODE	GO	Description	Log10(P)
	MCODE_1	R-HSA-909733	Interferon alpha/beta signaling	-67.6
	MCODE_1	R-HSA-913531	Interferon Signaling	-55.2
	MCODE_1	GO:0051607	defense response to virus	-42.1
	MCODE_2	R-HSA-418594	G alpha (i) signalling events	-12.8
	MCODE_2	R-HSA-500792	GPCR ligand binding	-11.4
	MCODE_2	R-HSA-373076	Class A/1 (Rhodopsin-like receptors)	-10.5
	MCODE_3	R-HSA-1474290	Collagen formation	-9.6
	MCODE_3	R-HSA-2022090	Assembly of collagen fibrils and other multimeric structures	-8
	MCODE_3	R-HSA-1650814	Collagen biosynthesis and modifying enzymes	-7.8
	MCODE_4	hsa04380	Osteoclast differentiation	-5.2
	MCODE_4	R-HSA-9658195	Leishmania infection	-4.9
	MCODE_4	R-HSA-9824443	Parasitic Infection Pathways	-4.9
	MCODE_5	R-HSA-1169408	ISG15 antiviral mechanism	-12.4
	MCODE_5	R-HSA-1169410	Antiviral mechanism by IFN-stimulated genes	-12.2
	MCODE_5	R-HSA-168928	DDX58/IFIH1-mediated induction of interferon-alpha/beta	-12.1
	MCODE_6	GO:0055086	nucleobase-containing small molecule metabolic process	-4.2
	MCODE_6	R-HSA-2262752	Cellular responses to stress	-3.8
	MCODE_7	GO:0045088	regulation of innate immune response	-4.7
	MCODE_7	GO:0002831	regulation of response to biotic stimulus	-4.4
	MCODE_7	GO:0045087	innate immune response	-3.8

Table S12. Enriched Metascape pathways using significant DEGs identified at week 24

Color	MCODE	GO	Description	Log10(P)
	MCODE_1	R-HSA-909733	Interferon alpha/beta signaling	-51
	MCODE_1	R-HSA-913531	Interferon Signaling	-41.8
	MCODE_1	GO:0051607	defense response to virus	-32.9
	MCODE_2	R-HSA-380108	Chemokine receptors bind chemokines	-17.6
	MCODE_2	hsa04062	Chemokine signaling pathway	-16.7
	MCODE_2	GO:0070098	chemokine-mediated signaling pathway	-16.5
	MCODE_3	R-HSA-1169408	ISG15 antiviral mechanism	-13.2
	MCODE_3	R-HSA-1169410	Antiviral mechanism by IFN-stimulated genes	-12.9
	MCODE_3	R-HSA-936440	Negative regulators of DDX58/IFIH1 signaling	-11.1

Table S13. Total number of NHP samples used for transcriptomics analysis

Adjuvant	Week 0	Week 0 + D2	Week 4 + D2	Week 12 + D2	Week 24 + D2
AH	7	7	6	6	7
ALFA	7	7	7	6	4
ALFQ	7	7	7	4	6
ALFQA	7	7	6	3	4
Total	28	28	26	19	21

Table S14. Pathways with antigen-specific CD8+ T cell frequencies for all five cytokines in the ALFQ arms

Time point	% of CD8	Direction	NAME	SIZE	NES	Bonferroni_adjusted P
W24D2	IFNg	Positive	ENRICHED_IN_MONOCYTES_(II)_(M11.0)	151	3.07	<0.001
W24D2	TNFa	Positive	ENRICHED_IN_NK_CELLS_(I)_(M7.2)	38	2.82	<0.001
W24D2	IL2	Positive	CELL_CYCLE_AND_TRANSCRIPTION_(M4.0)	275	2.73	<0.001
W24D2	TNFa	Positive	ENRICHED_IN_T_CELLS_(I)_(M7.0)	44	2.72	<0.001
W24D2	TNFa	Positive	CELL_CYCLE_(I)_(M4.1)	132	2.66	<0.001
W24D2	IFNg_and_IL2	Positive	B_CELL_SURFACE_SIGNATURE_(S2)	81	2.62	<0.001
W24D2	IL2	Positive	ENRICHED_IN_MONOCYTES_(II)_(M11.0)	151	2.61	<0.001
W24D2	IFNg_and_IL2	Positive	MEMORY_B_CELL_SURFACE_SIGNATURE_(S9)	20	2.49	<0.001
W24D2	TNFa	Positive	T_CELL_ACTIVATION_(I)_(M7.1)	42	2.45	<0.001
W24D2	TNFa	Positive	MITOTIC_CELL_CYCLE_DNA_REPLICATION_(M4.4)	24	2.40	<0.001
W24D2	IFNg	Positive	ENRICHED_IN_ACTIVATED_DENDRITIC_CELLS_(II)_(M165)	31	2.39	<0.001
W24D2	TNFa	Positive	T_CELL_ACTIVATION_AND_SIGNALING_(M5.1)	18	2.38	<0.001
W24D2	IL2	Positive	CELL_CYCLE_(I)_(M4.1)	132	2.38	<0.001
W24D2	IL2	Positive	DC_SURFACE_SIGNATURE_(S5)	70	2.36	<0.001
W24D2	IL2	Positive	ENRICHED_IN_ACTIVATED_DENDRITIC_CELLS_(II)_(M165)	31	2.35	<0.001
W24D2	TNFa	Positive	T_CELL_ACTIVATION_(II)_(M7.3)	26	2.34	<0.001
W24D2	IFNg	Positive	DC_SURFACE_SIGNATURE_(S5)	70	2.34	<0.001
W24D2	IFNg	Positive	MYELOID_CELL_ENRICHED_RECEPTORS_AND_TRANSPORTERS_(M4.3)	26	2.33	<0.001
W24D2	IFNg	Positive	TLR_AND_INFLAMMATORY_SIGNALING_(M16)	36	2.31	<0.001
W24D2	IFNg	Positive	ENRICHED_IN_MONOCYTES_(IV)_(M118.0)	47	2.28	<0.001
W24D2	IFNg	Positive	ACTIVATED_(LPS)_DENDRITIC_CELL_SURFACE_SIGNATURE_(S11)	33	2.26	<0.001
W24D2	IFNg_and_TNFa	Positive	ENRICHED_IN_NK_CELLS_(I)_(M7.2)	38	2.25	<0.001
W24D2	TNFa	Positive	MITOTIC_CELL_DIVISION_(M6)	27	2.24	<0.001
W24D2	IL2	Positive	ENRICHED_IN_MONOCYTES_(IV)_(M118.0)	47	2.23	<0.001
W24D2	TNFa	Positive	ENRICHED_IN_NK_CELLS_(II)_(M61.0)	10	2.22	<0.001
W24D2	IFNg_and_TNFa	Positive	PLATELET_ACTIVATION_(II)_(M32.1)	21	2.21	<0.001
W24D2	IFNg_and_TNFa	Positive	PLATELET_ACTIVATION_(I)_(M32.0)	22	2.20	<0.001
W24D2	IFNg	Positive	ENRICHED_IN_B_CELLS_(II)_(M47.1)	30	2.20	<0.001
W24D2	IL2	Positive	ACTIVATED_(LPS)_DENDRITIC_CELL_SURFACE_SIGNATURE_(S11)	33	2.20	<0.001
W24D2	IL2	Positive	PLK1_SIGNALING_EVENTS_(M4.2)	32	2.19	<0.001
W24D2	IFNg	Positive	ENRICHED_IN_B_CELLS_(I)_(M47.0)	36	2.17	<0.001
W24D2	IFNg_and_IL2	Positive	HOX_CLUSTER_III_(M17.2)	9	2.16	<0.001
W24D2	IFNg_and_TNFa	Positive	ENRICHED_IN_T_CELLS_(I)_(M7.0)	44	2.15	<0.001
W24D2	IFNg	Positive	TBA_(M66)	15	2.15	<0.001
W24D2	IFNg	Positive	MONOCYTE_SURFACE_SIGNATURE_(S4)	65	2.14	<0.001
W24D2	IFNg	Positive	ENRICHED_IN_B_CELLS_(IV)_(M47.3)	10	2.14	<0.001
W24D2	IFNg_and_IL2	Positive	DC_SURFACE_SIGNATURE_(S5)	70	2.13	<0.001
W24D2	IFNg	Positive	REGULATION_OF_SIGNAL_TRANSDUCTION_(M3)	41	2.12	<0.001
W24D2	IFNg	Positive	IMMUNE_ACTIVATION_GENERIC_CLUSTER_(M37.0)	256	2.11	<0.001
W24D2	IL2	Positive	TLR_AND_INFLAMMATORY_SIGNALING_(M16)	36	2.11	<0.001
W24D2	IFNg_and_IL2	Positive	EXTRACELLULAR_REGION_CLUSTER_(GO)_(M189)	13	2.10	<0.001
W24D2	IFNg_and_TNFa	Positive	CORO1A-DEF6_NETWORK_(I)_(M32.2)	16	2.07	<0.001
W24D2	IFNg	Positive	RESTING_DENDRITIC_CELL_SURFACE_SIGNATURE_(S10)	64	2.07	<0.001
W24D2	TNFa	Positive	T_CELL_SURFACE_SIGNATURE_(S0)	22	2.07	<0.001

W24D2	IFNg	Positive	INNATE_ANTIVIRAL_RESPONSE_(M150)	11	2.06	<0.001
W24D2	TNFa	Positive	MITOTIC_CELL_CYCLE_IN_STIMULATED_CD4_T_CELLS_(M4.5)	31	2.06	<0.001
W24D2	TNFa	Positive	CORO1A-DEF6_NETWORK_(II)_(M32.4)	13	2.05	<0.001
W24D2	IFNg_and_TNFa	Positive	T_CELL_ACTIVATION_AND_SIGNALING_(M5.1)	18	2.05	<0.001
W24D2	IFNg	Positive	TBA_(M55)	11	2.04	<0.001
W24D2	IL2	Positive	GROWTH_FACTOR_INDUCED_ENRICHED_IN_NUCLEAR_RECEPTOR_SUBFAMILY_4	10	2.02	<0.001
W24D2	IFNg	Positive	VIRAL_SENSING_&_IMMUNITY;_IRF2_TARGETS_NETWORK_(I)_(M111.0)	15	2.02	<0.001
W24D2	IFNg	Positive	TBA_(M72.1)	15	2.02	<0.001
W24D2	IFNg	Positive	CELL_CYCLE_AND_TRANSCRIPTION_(M4.0)	275	2.01	<0.001
W24D2	TNFa	Positive	PLATELET_ACTIVATION_(II)_(M32.1)	21	2.00	<0.001
W24D2	IL2	Positive	TBA_(M153)	13	2.00	<0.001
W24D2	IFNg_and_TNFa	Positive	T_CELL_ACTIVATION_(I)_(M7.1)	42	1.99	<0.001
W24D2	TNFa	Positive	MITOTIC_CELL_CYCLE_(M4.7)	20	1.98	<0.001
W24D2	IFNg	Positive	TBA_(M72.0)	20	1.97	<0.001
W24D2	IL2	Positive	TBA_(M72.0)	20	1.97	<0.001
W24D2	IFNg	Positive	ENRICHED_IN_NEUTROPHILS_(I)_(M37.1)	37	1.96	<0.001
W24D2	TNFa	Positive	T_CELL_DIFFERENTIATION_(TH2)_(M19)	13	1.96	<0.001
W24D2	IFNg	Positive	ANTIVIRAL_IFN_SIGNATURE_(M75)	21	1.95	<0.001
W24D2	IFNg	Positive	ENRICHED_IN_B_CELLS_(III)_(M47.2)	17	1.95	<0.001
W24D2	IFNg_and_TNFa	Positive	TBA_(M32.7)	10	1.95	<0.001
W24D2	TNFa	Positive	CORO1A-DEF6_NETWORK_(I)_(M32.2)	16	1.94	<0.001
W24D2	TNFa	Positive	E2F_TRANSCRIPTION_FACTOR_NETWORK_(M8)	11	1.93	<0.001
W24D2	IFNg	Positive	ENRICHED_IN_MONOCYTES_(III)_(M73)	11	1.93	<0.001
W24D2	IFNg	Positive	TBA_(M218)	11	1.90	<0.001
W24D2	IFNg_and_TNFa	Positive	T_CELL_ACTIVATION_(II)_(M7.3)	26	1.88	<0.001
W24D2	IL2	Positive	TBA_(M174)	19	1.87	<0.001
W24D2	IFNg	Positive	ENRICHED_IN_ACTIVATED_DENDRITIC_CELLS_(I)_(M119)	10	1.86	<0.001
W24D2	IFNg	Positive	ENRICHED_IN_MYELOID_CELLS_AND_MONOCYTES_(M81)	30	1.82	<0.001
W24D2	IFNg	Positive	TBA_(M79)	10	1.82	<0.001
W24D2	IL2	Positive	IMMUNE_ACTIVATION_GENERIC_CLUSTER_(M37.0)	256	1.78	<0.001
W24D2	IFNg	Positive	B_CELL_SURFACE_SIGNATURE_(S2)	81	1.75	<0.001

Table S15. Leading edge genes of the top ten enriched signatures

Signature	% of CD8	Leading_edge_genes
ENRICHED_IN_MONOCYTES_(II)_(M11.0)	IFNg	FBP1, SULF2, IRAK3, MYOF, TFEC, HNMT, CD86, PRAM1, CD36, TNS3, DMXL2, FES, NCF2, CYBB, LYN, CPPED1, KCTD12, LAT2, PLAUR, KYNU, CTSH, LRP1, CDA, CRISPLD2, PRKCD, TLR8, SYK, PLXNB2, S100A9, RBM47, LILRA1, S100A8, LILRA6, RASSF4, GCA, NOD2, ITGAM, RAB32, SLC15A3, IFI30, ITGAX, LILRA2, PTAFR, FGR, CYFIP1, SLC11A1, AQP9, HCK, CPVL, TNFAIP2, SPI1, CST3, RIN2, KLF4, CEBPD, NLRP12, MEGF9, HK3, ADAP2, SLC7A7, FPR1, CLEC7A, BTK, FCGR1A, IL13RA1, PILRA, LMO2, EPB41L3, CD163, FCER1G, TLR2, PLA2G7, AIF1, NCF1, PADI2, SGK1, P2RY13, ZNF467, PYGL, RXRA, CCR1, NLRP3, CSF3R, QPCT, MGST1, CSF1R, TGFBI, TREM1, FGL2, TLR5, MAFB, PLBD1, LILRA5, MPEG1, IGSF6, TLR4, RAB31, FCAR, TIMP2, ALDH1A1, CD1D, KCNE3, STAB1, C5AR1, GRN, KIAA0513, F13A1, PLXDC2, FPR2, AOAH, RTN1, MS4A4A, IMPA2
ENRICHED_IN_NK_CELLS_(I)_(M7.2)	TNFa	PLEKHF1, CLIC3, GZMM, CST7, NKG7, CD247, HOPX, PRF1, KLRD1, S1PR5, GZMA, PRKCQ, CCL5, SLAMF7, GIMAP7, NLRC3, STAT4, KLRF1, PRKCH, GZMB, ARL4C, FGFBP2, TGFBR3, SAMD3, IL2RB, KLRB1, RORA, TBX21, SH2D2A

CELL_CYCLE_AND_TRANSCRIPTION_(M4.0)	IL2	NDC80, KIF18A, CKS2, KIF20B, H2AFX, ZWINT, FBXO5, ESPL1, CCNA2, CCNF, DEPDC1B, NCAPH, IRAK3, CENPF, CENPE, HMOX1, CD86, HNMT, MYOF, CD36, TFEC, CCNB1, WDFY3, CDCA2, CPPED1, LILRA6, PRKCD, PLK4, NEK2, RRM2, OIP5, RACGAP1, LILRA1, NOD2, CENPA, KYNU, PLAUR, NCAPG, GPSM2, MARCKS, TNS3, EZH2, TOP2A, CDK1, MYBL2, CDC25C, FGL2, TTK, KIF23, RIN2, ECT2, FES, ANLN, FCER1G, DAPK1, HJURP, NCF2, SORT1, CDCA8, EXO1, TPX2, CEP55, SYK, DEPDC1, VCAN, KIF11, CDKN3, KLF4, S100A11, RBM47, CDCA7, CD163, MKI67, ARHGAP11A, CYBB, GMNN, DLGAP5, KIF4A, SEMA4A, PLK1, CDC20, APOBEC3B, E2F8, RRM1, FANCI, EPB41L3, AOAH, HCK, NUSAP1, IL13RA1, LRRC25, CD300LF, PILRA, DMXL2, ITGAX, FCGR1A, CCR1, CKAP2L, BRIP1, SLC11A1, IL1RN, MAFB, TLR2, RASSF4, HPSE, APOBEC3A, CEBPD, SPI1, CLEC7A, PLBD1, HMMR, SLC15A3, LILRA2
ENRICHED_IN_T_CELLS_(I)_(M7.0)	TNFa	PTPRCAP, CD3G, CD2, GZMM, NKG7, LCK, CD247, UBASH3A, PRF1, GZMA, PRKCQ, SIT1, CCL5, GIMAP7, NLRC3, CD7, PRKCH, GZMB, SH2D1A, GPR171, SAMD3, CD3D, KLRB1, CD6, RORA, CD3E, SLA2, TIGIT, CD28
CELL_CYCLE_(I)_(M4.1)	TNFa	GINS4, CENPH, POLA2, RFC3, CDCA3, RAD51, CENPM, TIPIN, CCNB2, NCAPD3, MAD2L1, ZWILCH, KIF20A, DTYMK, STMN1, PBK, CDT1, PRC1, CDC7, CDC6, HELLS, PCNA, SKA1, CDC25A, RFC5, KIF2C, MCM10, UHRF1, SPC24, E2F2, SPAG5, BRCA1, CENPK, DTL, SPC25, MELK, CDCA8, GINS2, DSCC1, CDC45, EXO1, SKA3, MCM5, MCM2, BUB1B, AURKB, KIFC1, BIRC5, NUF2, PTTG1, CDCA5, POLE2, MCM4, TYMS, CENPN, BRIP1, E2F7, HJURP, TPX2, SMC2, AURKA, FOXM1, KNTC1, NCAPG2, FEN1, KIF18B, E2F8, CENPW, RAD54L, NUSAP1, CCNE2, MKI67, POLA1, TUBG1, TACC3, MCM6, ASPM, CCNF, CDC25C, TIMELESS, SUV39H2, DLGAP5, GINS1, BUB1, KIF11, CDKN3, TRIP13, MYBL2, TTK, PLK4, ZWINT, CENPE, SMC4

B_CELL_SURFACE_SIGNATURE_(S2)	IFNg_and_IL2	SSPN, ITM2C, IL5RA, SCN3A, ROBO2, CDH5, SGCE, TMEFF2, CSMD1, GP5, PCDHB4, PCDHB10, PTPRK, HEPACAM2, CSPG4, ABCA8, HEPH, FREM2, CXADR, MOGAT2, NLGN1, F2RL3, CR2, SLC12A1, USH2A, LRP1B, IMPG2, NCAM2, NPY1R, NRXN3, EGF, SLC26A7, FLRT2, PCDH9, TSPAN13, CHL1, SLC6A16, TM4SF20, CADM2, POPDC3, SLC44A5, CXCR5, FCRL2, TRHDE, TLR10
ENRICHED_IN_MONOCYTES_(II)_(M11.0)	IL2	SULF2, MEGF9, IRAK3, FBP1, ITGAM, CD86, HNMT, MYOF, CD36, TFEC, CPPED1, LILRA6, PRKCD, LILRA1, NOD2, KYNU, PLAUR, TNS3, FGL2, CRISPLD2, LAT2, RIN2, CDA, FES, LYN, FCER1G, NCF2, SORT1, PRAM1, SYK, AQP9, RAB32, KLF4, S100A11, RBM47, CYFIP1, CD163, SGK1, CYBB, SMPDL3A, NLRP3, FCAR, EPB41L3, AOAH, HCK, MS4A4A, IL13RA1, PILRA, DMXL2, ITGAX, FCGR1A, CCR1, SLC11A1, MAFB, TLR2, PLA2G7, RASSF4, HPSE, S100A8, CEBPD, HK3, MGST1, SPI1, CLEC7A, PLBD1, SLC15A3, LILRA2, KCTD12, TLR4, KCNE3, CSF1R, FGR, IGSF6, MPEG1, RTN1, QPCT, TLR8, CPVL, CST3, S100A9, ALDH1A1, NLRP12, PLXNB2, SLC7A7, FPR1, PTAFR, PYGL, TNFAIP2, PADI2, TREM1, GCA, C5AR1, TIMP2, F13A1, CSF3R, TLR5, ADAP2, TGFBI, AIF1, CTSH, IFI30, LMO2
MEMORY_B_CELL_SURFACE_SIGNATURE_(S9)	IFNg_and_IL2	SSPN, ITM2C, TNFRSF13B, CSMD1, TNFRSF17, SORCS3, ABCA8, FREM2, CXADR, NLGN1, NCAM2, NPY1R, CHL1, CADM2
T_CELL_ACTIVATION_(I)_(M7.1)	TNFa	CORO1A, CD3G, CD2, CD8A, IL12RB1, LCK, CD247, PRKCQ, SIT1, CCL5, SLAMF7, NLRC3, CD5, CD7, PRKCH, IKZF1, CD3D, RORA, SLAMF1, CD3E, SLA2, TIGIT, CD28, ITPR3, IL21R, EOMES, CAMK4, BCL11B, ITK, TCF7, LEF1, DPP4
MITOTIC_CELL_CYCLE_DNA_REPLICATION_(M4.4)	TNFa	POLA2, MCM7, PRIM2, CDT1, CDC7, CDC6, PCNA, MCM10, MCM3, RPA2, CDC45, EXO1, MCM5, MCM2, POLE2, MCM4, TPX2, POLA1, MCM6
